# Tribute to Dr. Takuo Aoyagi, inventor of pulse oximetry

**DOI:** 10.1007/s00540-021-02967-z

**Published:** 2021-08-02

**Authors:** Katsuyuki Miyasaka, Kirk Shelley, Shosuke Takahashi, Hironami Kubota, Kazumasa Ito, Ikuto Yoshiya, Akio Yamanishi, Jeffrey B. Cooper, David J. Steward, Hiroshi Nishida, Joe Kiani, Hirokazu Ogino, Yasuhiko Sata, Robert J. Kopotic, Kitty Jenkin, Alex Hannenberg, Atul Gawande

**Affiliations:** 1 St. Luke’s International University, 3-4-2-3602 Toyosu, Koto-ku, Tokyo, 135-0061 Japan; 2 Department of Anesthesiology, Yale University, New Haven, CT USA; 3 Kyushu University, Medical Corporation Soseikai, Fukuoka City, Fukuoka Japan; 4 K and K Japan Co. Ltd, Tokyo, Japan; 5 Vital Sign Sensor Technology Development Division, Technology Development Operations, Nihon Kohden Corporation, Tokyo, Japan; 6 Department of Anesthesiology, Osaka University Medical School, Osaka, Japan; 7 Medical Equipment Division, Minolta Camera Co., Ltd. (Konica Minolta Co., Ltd., at present), Tokyo, Japan; 8 Department of Anesthesia, Critical Care and Pain Medicine Center for Medical Simulation, Harvard Medical School, Massachusetts General Hospital, Boston, MA USA; 9 Department of Anaesthesia and Pharmacology, University of British Columbia, Vancouver, Canada; 10 Tokyo Women’s Medical University, Tokyo, Japan; 11 Masimo & Patient Safety Movement Foundation, Irvine, CA USA; 12 Nihon Kohden Corporation, Tokyo, Japan; 13 Tokibo, Inc, Tokyo, Japan; 14 Critical Care Manager of Clinical and Medical Affairs, Edwards Lifesciences, Irvine, CA USA; 15 Lifebox Foundation, Brooklyn, NY USA

**Keywords:** Pulse oximetry, Patient monitoring, Anesthesia safety, History

## Abstract

**Introduction:**

Dr. Takuo Aoyagi invented pulse oximetry in 1974. Pulse oximeters are widely used worldwide, most recently making headlines during the COVID-19 pandemic. Dr. Aoyagi passed away on April 18, 2020, aware of the significance of his invention, but still actively searching for the theory that would take his invention to new heights.

**Method:**

Many people who knew Dr. Aoyagi, or knew of him and his invention, agreed to participate in this tribute to his work. The authors, from Japan and around the world, represent all aspects of the development of medical devices, including scientists and engineers, clinicians, academics, business people, and clinical practitioners.

**Results:**

While the idea of pulse oximetry originated in Japan, device development lagged in Japan due to a lack of business, clinical, and academic interest. Awareness of the importance of anesthesia safety in the US, due to academic foresight and media attention, in combination with excellence in technological innovation, led to widespread use of pulse oximetry around the world.

**Conclusion:**

Dr. Aoyagi’s final wish was to find a theory of pulse oximetry. We hope this tribute to him and his invention will inspire a new generation of scientists, clinicians, and related organizations to secure the foundation of the theory.

## Introduction

Katsuyuki Miyasaka, MD

### Preface

I feel greatly honored to have been asked by the previous Editor-in-Chief of *Journal of Anesthesia* (*JA*), Dr. Yamakage, to organize a special memorial issue on Dr. Takuo Aoyagi in English. I would like to thank him for his wise decision to take this timely opportunity to tell the world about the great contribution Dr. Aoyagi made to humanity. Many contributors are from countries other than Japan, all of whom showed great interest in this project. They kindly agreed to search their files and memories from 40 years ago and to send their memorial contributions on a tight schedule, so that we would have time to translate their articles into English. Furthermore, Dr. Yamakage has also contributed by editing them into an organized special article, which is fitting for an international journal, *JA*.

As you read through this collection of articles, many of you will find new information and developments previously unknown to the public. I hope that this memorial will give all of us the opportunity to reconfirm what we know of the great legacy left to us by the late Dr. Aoyagi.

### Oximetry: principle, but no theory

First, I would like to share with you one of Dr. Aoyagi’s final wishes. He always used to say “It is the role of researchers like me to establish a theory, but without a theory, there are limits to what devices can do. It concerns me that clinicians and society at large do not understand how this affects them.”

The pulse oximeter is a very special type of patient monitor that still, as of this date, has no standard method of calibration. It is generally thought that pulse oximetry simply measures photometric color changes of arterial blood. It is actually more complicated, as it measures changes in arterial blood color through dynamically changing filters, such as surrounding blood vessels, blood-filled tissue, bone and skin. Interference from these currently unpredictable, ever-changing factors, complicates the calculation of pulse oximetry measurement and it is difficult to separate the effects of these various factors on arterial blood oxygenation.

As an engineer and a developer, Dr. Aoyagi depended on clinicians like me to play an educational role and I believe that this memorial edition is important for letting people know about Dr. Aoyagi’s vision.

I am from a generation that experienced anesthesiology before the development of pulse oximeters. I was studying abroad in North America from 1973, when Dr. Aoyagi thought of the principle of pulse oximetry, until the end of 1977. This was just about the time when Minolta began selling a finger type device, and I knew nothing of Dr. Aoyagi’s existence or the idea of pulse oximeters. Japan was just starting to shed the image of Made in Japan = cheap and poorly made. The HP 8-wavelength ear oximeter was already in use at a research laboratory. Although it appeared accurate, it seemed to be cumbersome for clinical use.

It was not until 6 years after returning to Japan that I met Dr. Aoyagi at a Japanese Subcommittee of the International Organization for Standardization. We attempted, unsuccessfully, to establish a standardized calibration method. In the 36 years since I have had the privilege of learning from him and having lived through the same generation as a clinician and a developer, I feel a responsibility to record his achievements. I am most grateful for being entrusted with recording how his great invention was born and how it grew.

I hope that by translating the Japanese articles for this memorial into English, a wide range of medical professionals in the whole world will learn more about the birth and development of pulse oximetry. This memorial issue is being published to honor Dr. Aoyagi on the first anniversary of his death. Due to time constraints, the authors did not have much time to write, and I appreciate their cooperation. The authors, including me, are not historians and most never thought that they would be called upon to write a memorial article on short notice. There was not enough time to check for objectivity and consistency. There is no doubt that some uncertain statements, based on memories from long ago, are included. I hope you will understand the reasons for this and see that there is great meaning in publishing this compilation in terms of further development of pulse oximeters, just as Dr. Aoyagi wished. A short introduction to each of the authors can be found at the end of the compilation.

I am truly grateful to Dr. Yamakage for making the publication of this memorial issue possible in a timely fashion, the first year after Dr. Aoyagi’s death.

May Dr. Takuo Aoyagi rest in peace.

## The ratio of ratios and the Nobel Prize

Kirk Shelley M.D., Ph.D.

Professor Emeritus of Anesthesiology.

Yale University, New Haven, CT, USA.

I was truly heartbroken to hear of Dr. Aoyagi’s passing. His remarkable genius created the device that I have spent most of my life devoted to studying. He was a warm and gentle man who was profoundly humble. Shortly after the Yale symposium of the Innovations and Applications of Monitoring Perfusion Oxygenation and Ventilation conference (IAMPOV) in 2012, the Nobel Prize Committee approached me for a nomination for the 2013 Nobel Prize in Physiology or Medicine. This was done under an air of great secrecy. I wrote up Dr. Aoyagi’s nomination with care and thoughtfulness. I am disappointed to say that he was not given the award. To this day, I see this as my failure and not his.

When we met again face to face in 2015 in Tokyo at their IAMPOV. I took Dr. Aoyagi aside and broke the strict confidentiality rule. I explained to him that he had been formally nominated and considered for the Nobel Prize in Medicine in 2013. I apologized for the ineptness of my writing in being unable to convey the importance of the lifesaving nature of his work. He smiled warmly, thanking me most graciously.

Now that he has passed away, I am content that I violated my confidentiality agreement. He deserved to know how he came to be considered for the award and in what high regard people held him in. Below contains the essence and update of what I wrote in 2012. I have been subsequently invited to submit additional nominations over the years for other potential candidates. I find that I am unable to because of the sadness I feel at the passing of Dr. Aoyagi.

## A letter of recommendation

It was my pleasure and honor to nominate Dr. Takuo Aoyagi for the Nobel Prize in Medicine for 2013. My nomination was based upon his discovery of the “Ratio of Ratios”. That discovery is the core technology behind the modern-day pulse oximeter. In 1972, Dr. Aoyagi was interested in measuring cardiac output noninvasively by the dye dilution method using a commercially available ear densitometer. He concluded that it would require calibration, because arterial pulsatile “noise” prevented accurate recording of the dye clearance, and he invented a method to eliminate this noise, which led to his great contribution [1].

To quote Dr. Aoyagi [2], “These [pulsations] prevented accurate extrapolation of the down-slope of the dye curve after recirculation begins. I investigated this problem mathematically using the Lambert–Beer law. Then, I conceived the idea of eliminating the pulsation by computing the ratio of optical densities of the two wavelengths. This supposition was proved workable by experiments.”

Dr. Aoyagi goes on to say, “For this prototype, components of the dye densitometer were used. The light source was a small tungsten lamp. The transmitted light was divided into two beams, and each beam was received by a combination of an interference filter and a phototransistor. I used wavelengths of 630 nm and 900 nm. The wavelength of 630 nm was selected to maximize the hemoglobin extinction change caused by the oxygen saturation change, and the 900 nm wavelength was selected to avoid interference by the ICG dye. From the transmitted light intensity data, the pulsation amplitude AC and the total intensity DC were obtained, and the ratio, AC/DC, was calculated. This AC/DC ratio was obtained at both wavelengths, and their ratio, phi (Φ), was calculated. This is the so-called ratio of ratios. This phi (Φ) was supposed to correspond to SaO_2_.”

Dr. Aoyagi was correct; the ratio of ratios did correspond to the arterial saturation. The pulse oximeter has gone on to become a critical piece of medical equipment that is used all around the world on a daily basis. Its use is now considered to be a standard of care during surgical procedures and is part of a routine set of vital signs [3]. Its importance is further emphasized by the initiative, by the World Health Organization (WHO), called the “Global Pulse Oximetry Project”. This project was committed to the introduction of pulse oximetry technology throughout the world with an emphasis on developing countries [4].

The Nobel Prize committee has a tradition of awarding significant technical innovation in medicine. Willem Einthoven, in 1924 for his discovery of the electrocardiogram (ECG), Allan Cormack with Sir Godfrey Hounsfield, in 1979 for the development of computed tomography (CT) and Sir Peter Mansfield, in 2003 for his discoveries concerning magnetic resonance imaging (MRI) are such examples. I believe Dr. Aoyagi discoveries concerning pulse oximetry had achieved that degree of significance (Table 1).

**Table 1:** Short curriculum vitae of Takuo Aoyagi:

1958	Graduated electric engineering course of Faculty of Engineering, Niigata University Entered Shimadzu Corporation, Kyoto City as a researcher in the Central Research Laboratory
1966	Joined the Electronic Division, Faculty of Engineering, Tokyo University, where he studied cardiac output measurement using the dye dilution method
1971	Entered in Nihon Kohden Corporation, Tokyo City, where he engaged in the study of: < 1 > cardiac output measurement using dye dilution method < 2 > pulse oximetry: invention, making pilot model, and first announcement < 3 > pulse oximetry: constructing theory for improvement of pulse oximetry < 4 > pulse dye densitometry: for measurement of circulating blood volume < 5 > biomedical impedance method for lung monitoring
1993	Received a Ph.D. of Engineering at the Tokyo University, with a thesis topic of: "Study of noninvasive measurement of light absorbing materials in the blood using the arterial pulse."
1995	Achievement Prize by the Medical Instrument Society of Japan
2007	The Medal with Purple Ribbon (given by Japanese Emperor) Social Prize by the Japanese Society of Anesthesiologists
2012	The Harvey W. Wiley Lifetime Achievement Award
2013	J.S. Gravenstein Award Lifetime Achievement by Society for Technology in Anesthesia
2015	IEEE Medal for Innovations in Healthcare Technology

## Adoption of pulse oximetry into the JSA anesthesia safety guidelines—the brilliance of Aoyagi’s intellectual legacy

Shosuke Takahashi, M.D., Ph.D.

Professor Emeritus, Kyushu University.

Medical Corporation Soseikai, Fukuoka, Japan.

### Preface

When the concept of anesthesia for surgery was first introduced half a century ago, only one cardiograph was available at the operating theater, and we were checking for signs of crisis using this device in combination with visual inspection, palpation, percussion, auscultation, intermittent manual measurement of the blood pressure, and also clinical intuition. After pulse oximetry was introduced, it became possible to evaluate the status of oxygenation, ventilation, circulation, body temperature and muscle relaxation using scientific indicators, on the basis of the principle of vigilance. For example, it became possible to detect imminent cardiac arrest about 1 min before its occurrence if arterial blood oxygen saturation (SpO_2_) monitoring was performed, and several min before its occurrence if end-tidal carbon dioxide (ETCO_2_) monitoring was performed, whereas the time from the detection of signs of cardiac arrest to the occurrence of cardiac arrest was only 10 s when ECG monitoring was performed.

### Background

For us anesthesiologists whose mission is to protect the life of patients under conditions of body invasion, in which there may be only s/min left until the onset of a critical condition, introduction of the pulse oximeter enabling non-invasive measurement of SpO_2_ was a great relief, as if we had met Buddha in hell. This was a brilliant achievement which later triggered advances in the monitoring of vital signs as well. Discovery by Dr. Takuo Aoyagi, in 1974, of the principle of measurement of the arterial oxygen saturation using cardiac pulsations by pulse oximetry has contributed greatly to mankind. At first, an ear oximeter was manufactured on a trial basis for clinical application of this principle, but the device was not commercialized, and research for its commercialization suffered a setback and delay.

Nearly, 10 years later, pulse oximeters with fingertip sensors were developed in the US, followed by the rapid spread of these devices. Japan hastily introduced the devices made in the US without laying claim to the fact that the pulse oximeter principle was first invented in Japan (by Dr. Aoyagi), until Prof. Severinghaus (University of California, San Francisco) publicized the fact in 1986.

I was shocked when I was informed by Prof. Michiaki Yamakage (Chief Secretary, Japan Association for Clinical Monitoring) of the death of Dr. Takuo Aoyagi on April 18, 2020. At that time, my memory of him ran like a revolving lantern, including my seeing Dr. Aoyagi, while he was alive, and events related to him during the days when I was involved in spreading the clinical application of pulse oximeters. On this occasion, I would like to present some of those scenes in memory of Dr. Aoyagi while dedicating my deep respect and gratitude to him.

### Operating room safety management committee

It was in 1976 that the Operating Room Safety Management Committee was constituted within the Japanese Society of Anesthesiologists (JSA). Prof. Hideo Yamamura (University of Tokyo) served as the first chairman of this committee. Later, the role of the chairman was passed on to Professors Toyohisa Arai, Keizo Takahashi, Ken’ichi Kobayashi and Masahiro Suzuki in that order, and the baton was also handed at one time to the author. In those days, anesthesia was viewed unfavorably by people, and there prevailed a negative attitude about anesthesia itself being a risk factor for operation. For example, when the patient died as a result of a poor operative outcome, explanation like the following was often offered without hesitation: “The operative procedure itself was successful, but the patient failed to wake up from anesthesia,” etc.

On the 31^st^ Conference of the JSA in 1984, a symposium titled “Towards Safer Anesthesia” was organized. This was the first meeting focusing on the safety of anesthesia in the setting of an academic conference. Around that time, I began to place great importance to the activities within the framework of the Operating Room Safety Management Committee, which eventually led to the publication of “Guidance on Monitoring for Safe Anesthesia” on April 21, 1993. The process until preparation of this guidance is described in the published monograph “Guidebook—Guidance on Monitoring for Safe Anesthesia by the JSA” (edited by Masahiro Suzuki and Toyohisa Arai, published by Kokuseido Co., Ltd., 1995). This guidance has been revised four times and provides easily understandable guidance while avoiding ambiguous expressions.

A survey of accident cases related to anesthesia was conducted in parallel with the creation of this guidance. At some point of time, some of the survey staff proposed that the survey be discontinued, because data collection and entry were labor-intensive. However, our definitive determination to continue with the survey was understood by successive members of the committee and the staff in charge of the survey at each leader hospital, so that the survey has been continued to date, without interruption. Analysis of the data collected during this survey has yielded significant outcomes, such as the creation of the guidance for countermeasures against massive bleeding and guidance for prevention of pulmonary embolism.

### Why was the guidance created so rapidly?

Development of a concrete guidance was started at a time when standards on intraoperative monitoring were being published one after another in European countries, modeled after the standard published in 1986 by the American Society of Anesthesiologists (ASA). Needless to say, the introduction of pulse oximetry served as a driving force for these actions.

Another factor which prompted rapid creation of the guidance was the death of two patients caused by accidental erroneous inhalation of pure nitrous oxide at an influential national hospital in Kyushu in 1987. The episode was attributed to an error in the arrangement of the supply pipes for oxygen and nitrous oxide made during the construction of the hospital. Although the anesthesiologists involved in the care of the victims were exempted from legal responsibility, a tense atmosphere prevailed in those days at the mention of anesthesia, and we resolved to never have such accidents recur.

### Preparation for wiping out the demon: Monitored Anesthesia Care (MAC)

In recent years, it has become mandatory for the guidance on monitoring to be followed if a test or treatment requiring anesthesia is undertaken even outside the operating room. Introduction of such a regulation is also being debated now in Japan, under the so-called MAC. If anesthesia provided without the MAC results in an adverse event, the healthcare provider concerned will be judged as “having been negligent, i.e., there is a default obligation on experts to predict the possible risks associated with a given medical act and to take steps to avoid the risks.” Simply said, invasive medical acts under environments that lack preparation for risk management are unacceptable. MAC is “a preparatory step for wiping out the demon” hidden behind medical acts. In Japan, however, reimbursement for MAC is not provided by the national health insurance system to the physicians in charge of vigilance, putting a considerably heavy burden on the healthcare providers.

### Japan association for clinical monitoring

When talking about Dr. Aoyagi, I cannot omit to referring to the Japan Association for Clinical Monitoring founded under the initiative of Prof. Akira Okuaki (Fukushima Prefectural Medical University). At the meetings of this association, Dr. Aoyagi often presented research data aimed at improving the precision of pulse oximeters and optimizing the cost of this device. He also made a presentation at the 7th conference of this association held in Fukuoka and was awarded the Okuaki Memorial Prize in the following year. In those days, Prof. Katsuyuki Miyasaka (National Children’s Hospital) was also actively involved as a co-researcher in the research conducted by Dr. Aoyagi. Prof. Okuaki invited researchers from varied specialties to this association to allow multidisciplinary wisdoms to debate an issue, and Dr. Aoyagi was a very valuable asset for this association. It was also impressive that Prof. Kunio Suwa (University of Tokyo), who was the best speaker on oxygen-related topics among Japanese researchers, enthusiastically suggested that the performance of Dr. Aoyagi was worthy of a Nobel Prize.

### Talk of Nobel Prize

#### Awarded to the research on hypoxia-inducible factor (HIF)

The year 2001 was the 100th anniversary of awards of the Nobel Prize. In those days, Prof. Lindahl (Department of Anesthesiology, Karolinska Institute) was the chairman of the Nobel Committee for Physiology or Medicine. I became acquainted with him in those days, and we became reasonably good friends as we were around the same age (born in 1943). Prof. Lindahl always talked passionately about the beauty and novelty of science.

We asked him to deliver a special speech at the 49th Conference of the Japanese Society of Anesthesiologists in 2002. We asked him to refer to the (1) criteria for selection of a Nobel Prize winner and (2) introduction of the research in our field that would be the worthiest of this prize, when delivering the special speech. He immediately responded to this request, saying that the answer to (1) was “good for mankind”, i.e., great contribution to mankind, and to (2) was the research on biological reactions to hypoxia from the standpoint of molecular genetics. On that day at the Conference, his audience was fascinated by his smart and appealing special speech.

The Nobel Prize in Physiology or Medicine 2019 was awarded to 3 researchers, including Prof. Semenza (Johns Hopkins University), for their contribution to elucidation of the mechanism of cellular sensing and responses related to oxygen utilization. We were excited much by the Nobel Prize having been awarded for a field of research that was of interest to us. According to the special article by Prof. Kiichi Hirota (Kansai Medical University), published in the November issue of LiSA, 2019 in Japanese, these 3 researchers were awarded the prize for their detection and isolation of HIF as a factor, a component of the molecular mechanism involved in the induction and maintenance of erythropoietin expression, as well as elucidation of the molecular mechanism for oxygen tension-dependent HIF activity modulation. I hear that Prof. Hirota was involved in isolating the gene encoding the HIF molecule as a member of the laboratory led by Prof. Semenza.

Very broadly speaking perhaps, the connection between SpO_2_ and HIF can be viewed as a dialogue between oxygen and the living body during the course of external and internal respiration closely involved in homeostasis and evolution.

#### Criteria for award of the Nobel Prize

During the 75 years after World War II, slightly more than 25 Japanese have been awarded the Nobel Prize. Why was Dr. Aoyagi not awarded the Nobel Prize? Prof. Kunio Suwa had pointed out on several occasions that Dr. Aoyagi deserved this prize, and Prof. Katsuyuki Miyasaka had also provided strong support for the awarding of this prize to Dr. Aoyagi. I also tried to use the best of my limited abilities for this purpose, advising Prof. Lindahl (Chairman of the Nobel Committee) that the performance of Dr. Aoyagi satisfied the criterion “good for mankind” for award of the Nobel Prize. At that time, I thought the chairman subtly told me that while his work was great, it was still some distance from deserving the Nobel Prize. However, because of my poor language abilities, I am not confident that I understood his response completely. He did seem, though, to suggest the weak points of Dr. Aoyagi, i.e., the fact that his first paper was written in Japanese and that the intellectual proprietorship of the outcome of his research had not been established by a globally valid method. It has been pointed out for many years that the basis for the protection of intellectual proprietorship is weak in Japan and that Japanese enterprises show poor capability for translating advanced technologies. I think that these shortcomings remain open issues even until date.

Despite such limitations, the greatness of Dr. Aoyagi’s performance has been steadily enriched. His research has led to submission of many applications for patent registration (linked to US patent registration), publication of papers in journals, such as Anesthesiology and Anesth Analg, and awards of many professional society prizes, such as the Patent Agency Director’s Encouraging Prize, the Science and Technology Director Award, the Medal with Purple Ribbon, Gravenstein Lifetime Achievement Award of the Society for Technology in Anesthesia (USA), the Institute of Electrical and Electronic Engineers (IEEE) Medal For Innovations In Healthcare Technology, and so on. Thus, global recognition of the performance of Dr. Aoyagi has been deepening. Although not satisfying the criteria for the Nobel Prize, the intellectual relics left by Dr. Aoyagi will remain brilliant forever.

## Different roles of Japanese and US industry in the clinical introduction of pulse oximeters

Hironami Kubota.

Medical devices market research consultant.

K and K Japan Co. Ltd., Tokyo, Japan.

### Long way from development to commercialization

The invention of the pulse oximeter by Dr. Takuo Aoyagi in 1974 has been viewed as a typical case of medical device development achieved under a plan based on definite goal setting. The favorable outcome of such an approach to development is shown by the fact that the medical device developed thus has been commercialized and adopted widely across the globe. In the medical device industry, characterized by small-lot large-variety production, scarcely any medical device has contributed as significantly to healthcare as the pulse oximeter. Thus, it may be called “a good model of medical device development.”

I have just given the conclusion at the beginning of this paper. When the project led by Dr. Aoyagi was under way, I was also a member of the Development Department of Nihon Kohden Corporation. In those days, I could hardly have imagined that the device development under way at the neighboring section would advance to such a remarkable extent, because the missions of different sections of the department differed completely from each other. Neighboring sections were expected to make efforts together for improvement through friendly rivalry. The job that I was in charge of in those days pertained to improving patient monitors (e.g., adoption of wireless monitor systems), which had already advanced to a certain degree, and this mission was different in nature from that of in which Dr. Aoyagi was engaged. In other words, the topic of development assigned to my section, which pertained to improving the practical aspects of existing products, differed in dimension from the innovative development topic assigned to the Aoyagi Project.

“Optical Oxygen Measuring Device,” application for patent registration was filed in 1974 in Japan. This device later served as the prototype of the pulse oximeter. To this document, the memo “Registration NO. 947,714” was later added manually. This memo is a valuable relic written by Dr. Aoyagi himself. A noteworthy phrase used in this bulletin is the “earpiece-type oximeter” as an example of patent embodiment. This was commercialized as the initial product of an “ear oximeter.” Here, the term “oximeter” means “measuring oxygen” and the phrase “earpiece-type” indicates “measurement attaching to the earlobe.”

From the standpoint of engineering, the ear oximeter was characterized by the use of an analog device, with utilization of primitive electric parts like light bulbs as the source of light. It is an undeniable fact that these characteristics of the ear oximeter led to unstable of the measurements, resulting in the development of the domestic impression that the product was “useless.”

Efforts to overcome such a challenge were made by Minolta Camera, Inc. (currently named “KonicaMinolta, Inc.”). OXIMET, which was the first oximeter marketed by Minolta Camera, was characterized by the utilization of a “finger sensor”. It is noteworthy that this product for routine use was marketed only several years after the ear oximeter was launched in the market. Considering that most pulse oximeters currently on the market adopt this type of sensor, we may say that the OXIMET served as the driving force for establishment of the path towards development and commercialization of pulse oximeters for routine use.

However, the clinical usefulness, in the true sense of the term, of pulse oximeters became apparent only after the products were marketed by two American companies. The term “pulse oximeter” became popular only at in those days. This is because this kind of device was shown to play a significant role in the fields of anesthesiology/critical care and neonatal management.

Table 2 summarizes the course of pulse oximeter development, focusing on the actions/events in Japan and the USA. It can be seen from this table at a glance that the efforts made in Japan primarily pertained to the principle and development, while those in the USA contributed to implementation of pulse oximeters. On the basis of the efforts made in these two countries under such role assignment, pulse oximeters have so far grown into a group of devices that are used extensively in routine clinical practice around the world.

**Table 2:** Course of pulse oximeter development—comparison between Japan and the US

Year	Role of Japan	Role of the US
1974	Patent application for the pulse oximetry principle filed by Takuo Aoyagi (Nihon Kohden)	
1977	Pulse oximeter marketed by Akio Yamanishi et al. (Minolta Camera)	
1981		Pulse oximeter (the first full-scale product) marketed by Nellcor and Ohmeda
1992	International Standard on Pulse Oximeter ISO 9919 enforced	
1994	Wrist watch-type pulse oximeter marketed by Techna Electronic Industry (currently named “T & RK”)	
1996		Fingertip-type pulse oximeter marketed by Nonin
1998		Software (Signal Extraction Technology) for improving the precision of measurement with pulse oximeters developed by Masimo
2000 ~	Portable/wearable products developed by many manufacturers, including Nihon Seimitsu-sokki, Inc	
2005		Pulse oximeter allowing measurement under the environment for MRI marketed by Nonin
2011	International Standard on Pulse Oximeter ISO 800,601–2-61 (revision of ISO 9919) enforced	
2014	Domestic standard on pulse oximeter JIS T80601-2–61 enforced	
2020		“Blood Oxygen Wellness” introduced into common products by Apple Inc

### Biased towards the two extremes (high-functioning products vs. extensively applicable products)

Pulse oximeters that were successfully commercialized by the two American companies Nellcor and Biox (Ohmeda) as products for routine use were both standalone-type (Type A) products and were intended primarily for use in critical care (intraoperative care in severely sick patients, premature infant/neonate management, and so on). Development of these products was led by Prof. Severinghaus of the University of California, San Francisco, who emphasized the significance of continuous monitoring of arterial blood oxygen saturation enabled by pulse oximeters. The contribution of Prof. Severinghaus is particularly consequential, in that he positioned a pulse oximeter as an optimal device for use in intraoperative respiratory management and thus, as an indispensable device for anesthesia management. At that time, he announced to the world that this principle related to a pulse oximeter was actually discovered by Dr. Takuo Aoyagi of Japan [5]. It was Dr. Kunio Suwa (Department of Anesthesiology, University of Tokyo, in those days) who actively disseminated this information in Japan. This information, announced ten-odd years after registration of the related patent in Japan, was perceived as “information that had never been heard before” or “information that should prompt re-recognition,” even in Japan, the country where the researcher originated from.

Thereafter, new types of pulse oximeters were marketed one after another, not only by American manufacturers, but also by Japanese and European manufacturers. In the 1990s, pulse oximeters became general-purpose devices, applicable also to the care of moderately severe patients and in general wards. In the 2000s, technological advances in pulse oximeters became accelerated, resulting in the development of compact-sized/portable products and wearable products (Type B), thereby enabling more extensive use of pulse oximeters. Among others, spread of the fingertip sensor-combined type of pulse oximeter generated a new role for the pulse oximeter (a spot checker of oxygen saturation in ordinary patients and in health management in general; Type B), in addition to its previous role of patient monitoring; Type A.

The standalone-type pulse oximeters (Type A) mainly consist of high-functioning products often made overseas (e.g., USA). An example is a pulse oximeter that offers improved precision of measurement and is “capable of dealing also with bodily movements” [6] or “enabling measurement even during hypoperfusion.” There are also products which have been categorized as “pulse photometry” products that are capable of measuring abnormal hemoglobin (carbon monoxide-bound hemoglobin, methemoglobin, etc.), in addition to measuring the oxygen saturation level.

Among the Type B products, Japanese products are predominant. While typically, it is difficult for big businesses to enter this market, the entry by venture companies, such as small- and medium-sized enterprises, is rather remarkable; as many as more than 30 such companies have entered this market in Japan, with Japan Precision Instruments, Inc., serving as the market leader. This device is fitted with a wireless communication function and is linkable to smartphones and clouds, reflecting the latest trends in the field of information technology.

### Essential nature of pulse oximeters and its relationship to international standards

Now, the term “pulse oximeter” is known well; people hearing this word are likely to imagine a compact-sized Type B device. However, during the stage of development and early phase of commercialization, i.e., until the 1980s, only the full-scale type (Type A) of pulse oximeters were available in the market. After the turn of the century, however, this market changed dramatically, and Type B products have become predominant. Now, I shall refer to open issues related to Type B pulse oximeters, the currently predominantly used type of oximeter.

My greatest concern pertains to the large discrepancy between the international standards and the current status of Type B pulse oximeters available in the market. The current international standard ISO80601-2–61: 2011 was set forth, covering Type A full-scale pulse oximeters as the main target. For example, the section about precision of measurement in this standard requires implementation of a study involving strict comparison between the arterial oxygen saturation (SaO_2_) measured by the oximeter and the SaO_2_ in the patient’s arterial blood (in arterial blood samples) in healthy volunteers under induced hypoxemia. This means that even though a pulse oximeter is “essentially a non-invasive device,” the standard requires that it provides clinical evaluation results equivalent in precision to those of “highly invasive devices” of a different nature.

When viewed from the angle of underlying principle or the design concept during development, the primary principle of a pulse oximeter is “(non-invasive) extracorporeal measurement of oxygen saturation with the use of light.” It is no exaggeration to say that this principle or concept served as the basis for the global spread of this type of device. Considering that the predominantly used type of pulse oximeters changed from Type A monitors to the current compact-sized type monitors, it is understandable that a large discrepancy arose between the international standard and the actual status of type B pulse oximeters. However, a fundamental issue is that no ideal standard criterion device for “non-invasive” calibration of a pulse oximeter is available until date.

There is another factor that needs to be borne in mind. In Japan, the ISO standard mentioned above was translated directly, to yield JIST80601-2–61:2014. Then, this part of the JIS was cited directly in the pulse oximeter accreditation criteria set forth pursuant to the Pharmaceutical and Medical Devices Act. This poses a problem. Originally, each provision of the international or domestic standards serves as “a standard” and cannot be interpreted as indicating “must” (mandatory). However, if such a provision of the international or domestic standards is incorporated directly into the accreditation criteria based on the Pharmaceutical and Medical Devices Act, the provision suddenly changes to assume the meaning “must” (mandatory). We see a possibility that those involved in legislation of the law were not aware of this hidden mechanism.

Thus, the issue has two aspects: (1) the standard available at present does not match up to the current status and (2) the law has carelessly converted the “standard” into a “mandatory rule.” I fear that although the pulse oximeter has grown in stature to one of the most frequently used medical devices in the world, a lack of sufficient understanding of this device by the regulatory authorities/administrative organs can hamper or markedly delay new development and/or clinical introduction of pulse oximeters for medical use.

### Relationship to IT and perspectives for future

The spread and expansion of the use of pulse oximeters across the world are showing no signs of stopping or slowing down. This can be viewed as an outcome of the efforts made to date by manufacturers around the world towards “commercialization” of their products while adopting new ideas/designs and competing with each other. In other words, “rivalry/free competition” serves as the basis for device improvement, and this should be viewed as a very favorable direction of development. Furthermore, interlinking with the latest IT-related devices in terms of technology and merchandise has also been achieved, resulting in elevation of the value of pulse oximeters as a merchandise.

For example, the new concept of “Blood Oxygen Wellness” was introduced into Japan, in September 2020 to the Apple Watch 6. The function and performance of this watch are estimated to be approximately comparable to those of existing pulse oximeters in terms of the technique and principle of measurement. A difference lies in that existing terms such as “pulse oximetry” and “SaO_2_” are not used in this watch, and this product has been viewed as a product reflecting the manufacturer’s intention of creating the new concept of healthcare or wellness devices derived from the existing concept of medical devices. I have already stated that from a historical point of view, pulse oximeter use is shifting from Type A to Type B devices. Apple Watch 6 should be classified as another new type of device. From an industrial standpoint, this product may be viewed as creating a new field and can serve as a starting point for arguments over the form and nature of devices used for health promotion and management from now on. However, from the legal point of view, no statement has been issued by the regulatory authorities like the Pharmaceutical and Medical Devices Agency (PMDA) in Japan or the approval/accreditation system of Food and Drug Administration (FDA, USA) about approval/accreditation of this type of product.

We may expect further sophistication in the software used for pulse oximeters in the future. For example, research is now under way over measurement of the respiratory rate from changes in the pulse wave, and so on. In addition, further efforts will be made to explore the possibility of improving the precision of oxygen saturation measurement using a multiple wavelength system (a topic to which Dr. Aoyagi dedicated his passion), adding the capability for measuring other parameters, and so on. Contactless sensors for use in pulse oximeters are also under development and their commercialization in the near future is highly probable.

In the end, I would like to add that the heritage left by Dr. Aoyagi (given the shining title of “Father of Pulse Oximeter”) and his guidance on new research and development give us a strong impression that pulse oximeters will continue to evolve in the future.

## Using a fundamental approach to improve the accuracy of pulse oximetry—the research to which Dr. Aoyagi devoted his life

Kazumasa Ito.

Vital Sign Sensor Technology Development Division, Technology Development Operations.

Nihon Kohden Corporation, Tokyo, Japan.

### Introduction

Around 2007, Dr. Aoyagi once attended an in-company lab meeting with a newspaper clipping. The article reported a medical accident due to a missed alarm. At the very beginning, he insisted, “The performance of the device is the root cause. Performance improvement is essential in medical settings. We have to improve measurement technologies to reduce medical accidents.” He showed a strong sense of mission, saying, “fundamental improvement in the performance of pulse oximeters by constructing a theory not only allows a reduction in the burden on patients of blood collection, but also leads to the prevention of alarm-related accidents due to health care workers’ alarm fatigue [7]. Furthermore, it widens the possibility of application contributing to health care, as a method of noninvasive and continuous measurement of light-absorbing materials in blood.”

Today, devices with closed-loop control function have begun to be actively used, and the level of accuracy required for pulse oximeters is changing. From the beginning, Dr. Aoyagi had a clear vision that “the ultimate ideal of health care is automatic control of treatment,” and he worked actively to improve the performance. In this article, I introduce Dr. Aoyagi’s research on theory construction by multi-wavelength pulse oximetry.

### Accuracy of pulse oximetry

First, I would like to explain the accuracy of pulse oximetry, which Dr. Aoyagi pursued throughout life. Although products currently on the market have an accuracy of approximately ± 2% SpO_2_ according to specifications, it should be noted that while two-thirds of the SpO_2_ values are within the range of accuracy specified, one-third are outside the range, and that the range does not correspond to the 95% confidence interval. In addition, hypoxia exposure testing to verify the accuracy is performed under almost ideal conditions in healthy subjects, and various factors may further decrease accuracy in the actual clinical environment.

Dr. Aoyagi cited oxygen management in neonates as an example and set the goal of realizing true second-generation pulse oximetry with increased absolute accuracy, to reduce the patient burden by preventing retinopathy of prematurity and reducing the number of blood collections.

### A challenge to fundamentally improving the accuracy

Dr. Aoyagi recognized that there was room for improvement in the accuracy of pulse oximetry soon after the dissemination of two-wavelength pulse oximeters in Japan. A theory considering the effects of changes other than those in the arterial blood is required to improve the accuracy of pulse oximetry. Changes other than those in the arterial blood itself are caused by body motion and other factors, and he started a study based on the fundamental idea that the construction of this theory would lead to the elimination of body motion artifacts and provide a basis for significant future development.

In products of Masimo Corporation, which is a pioneer of algorithms with improved body motion-resistance performance, the most provable oxygen saturation is displayed as the SpO_2_ value by performing all calculations in the oxygen saturation range of 1–100%. Other companies have also adopted technologies to separate components corresponding to arterial oxygen saturation by extracting the characteristics of the pulse wave, but either technology involve assumptions. In contrast, Dr. Aoyagi tried to express all phenomena affecting the accuracy of pulse oximetry through mathematical formulae (i.e., to fix the theory). He tried to fundamentally improve the accuracy of pulse oximetry, with the belief that if all the ongoing phenomena can be explained, an accurate SpO_2_ value can be obtained.

**Fig. 1:**
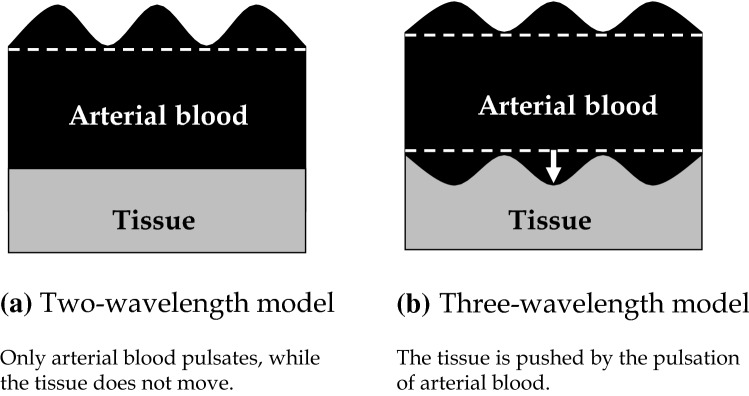
Models of principles of pulse oximetry.** a** Two-wavelength model. Only arterial blood pulsates, while the tissue does not move.** b** Three-wavelength model. The tissue is pushed by the pulsation of arterial blood

In the course of research, Dr. Aoyagi paid attention to the Lambert–Beer law, which is the basis for two-wavelength pulse oximetry. According to the law, the attenuation of light entering a medium (degree of optical attenuation) is proportional to the extinction coefficient, which indicates how much light a medium absorbs, and the thickness of the medium if the medium is homogeneous. Oxygen saturation is supposed to be easily measured according to this law on the assumption that the pulsatile component of light transmission through living tissues is caused by light absorption of arterial blood (Fig. 1a two-wavelength model). However, practically, the degree of optical attenuation is not simply proportional to the extinction coefficient and the change in thickness, because it is affected by complex factors including not only light absorption, but also light scattering. Since the theory has not been established, numerical values are displayed according to a look-up table based not on theoretical calculations, but on measured values obtained from subjects under severe hypoxic conditions. This method is invasive and reflects the reality in which there are no standardized calibration methods. Establishment of a theoretical formula will lead not only to improved accuracy and elimination of the effects of body motion, but also to the establishment of a standardized calibration method, which is not currently available.

Although light scattering was an unfamiliar field for Dr. Aoyagi, he worked energetically and tried to make improvements in the following order:

- 1) To establish a theoretical formula for light absorption/scattering, including error factors (Schuster theory adopted for scattering [8]).
- 2) To increase the number of light wavelengths and to equalize the number of equations for the attenuation ratio between two wavelengths (Φ) to the number of unknowns.
- 3) To set up simultaneous equations for Φ.
- 4) To obtain accurate SpO_2_ values from the simultaneous equations.

### Consideration of the effects of tissues

**Fig. 2:**
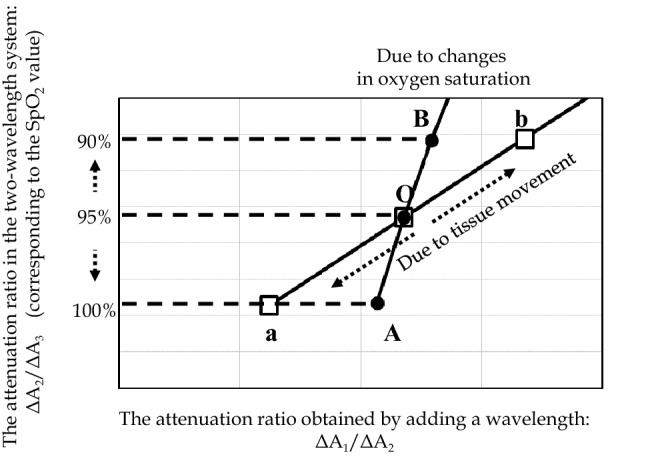
Simulations considering the effects of tissues. Changes in the attenuation ratio when tissue pulsation is absent and only oxygen saturation changes. Changes in the attenuation ratio when oxygen saturation is constant and the tissue thickness changes

Dr. Aoyagi first paid attention to non-blood living tissues as a significant factor other than arterial blood. When the effects of tissues are taken into account, arterial oxygen saturation and the effects of tissues are variable factors. Dr. Aoyagi obtained two attenuation ratios by measurements at three wavelengths to allow separation of the effects of changes in the arterial blood and changes in the tissues (Fig. 1b three-wavelength model). Attenuation changes by ΔA_i_ at each wavelength λ_i_ (i = 1, 2, 3) depend on the changes in the arterial oxygen saturation and tissue thickness, and Fig. 2 shows the results of the simulations on the assumption of one of the two changing. The vertical axis represents the attenuation ratio of the two wavelengths used in conventional pulse oximeters ΔA_2_/ΔA_3_ (corresponding 1:1 to the SpO_2_ value), and the horizontal axis represents the attenuation ratio ΔA_1_/ΔA_2_ obtained using an additional wavelength (λ_i_). The line passing through points A and B represents the change in the attenuation ratio when the tissue thickness does not change and only the oxygen saturation changes, while the line passing through points a and b represents the change in the attenuation ratio when the oxygen saturation remains constant, and the tissue thickness changes. Although the oxygen saturation is different between points A and a, the two-wavelength system cannot distinguish the difference and displays the same oxygen saturation. It also cannot distinguish the difference between points B and b. Measurements at three wavelengths allow accurate determination of the oxygen saturation corrected for the effects of changes in the tissue thickness.

**Fig. 3:**
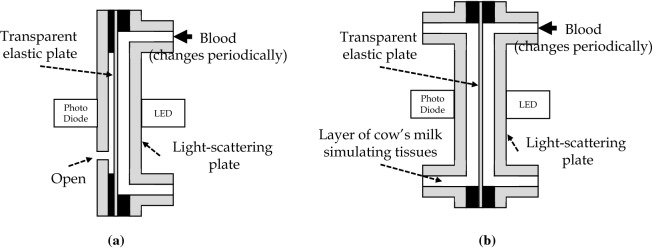
In vitro experimental models.** a** Single-layer model (only arteries change). Changes in the blood layer reduces the thickness of the open air layer. **b** Two-layer model (the effects of tissues were simulated). Changes in the blood layer reduces the thickness of the layer of cow’s milk (tissues). The thickness of the blood layer is changed periodically by changing the pressure of blood using a transparent elastic plate on one side of the blood layer.

**Fig. 4:**
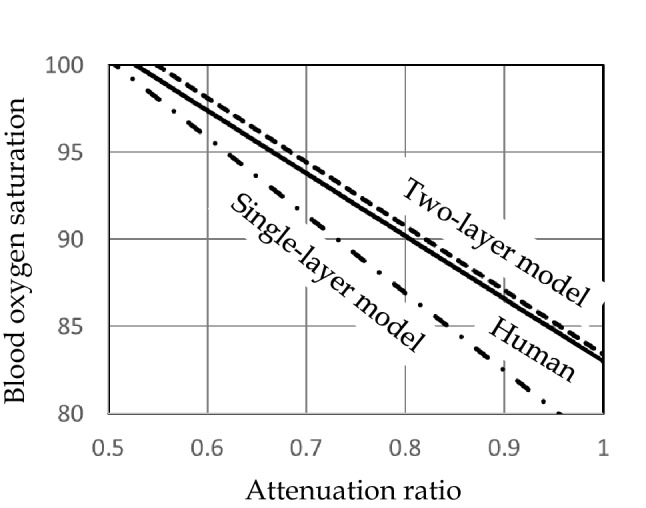
Comparison of measured values between the in vitro models and humans [13]. The measurement results in the two-layer model tended to be more closely similar to those in humans than those in the single-layer model.

At first, after his proposal of the theory in relation to tissues, this theory was not easily understood by people around him, due to the complicated behavior of light in vivo. However, Dr. Aoyagi confirmed his theory and explained it with conviction by the following procedure. He investigated the effects of tissues by in vitro experiments using cuvettes (thin cells). Using a single-layer model in which only the arteries change (Fig. 3a) and a two-layer model in which tissues are simulated by a layer of cow’s milk (Fig. 3b), the attenuation ratio was measured when blood oxygen saturation in the cuvette was changed to compare the relationship between the oxygen saturation and the attenuation ratio with that in human subjects (Fig. 4). The results showed that the relationship between the oxygen saturation and the attenuation ratio in the single-layer model was different from human data that in the two-layer model was consistent with human data. He confirmed that the effects of tissues are a major error factor and concluded that the accuracy can be improved by wavelength multiplexing.

### Consideration of the effects of veins

To further improve the accuracy, Dr. Aoyagi paid attention to the effects of venous blood in addition to those of tissues. Although it was difficult to construct a theory considering venous blood, he believed that the accuracy can be fundamentally improved by constructing a five-wavelength model considering the following four factors with optical effects.

- 1) Effects of arterial oxygen saturation.
- 2) Effects of venous oxygen saturation.
- 3) Effects of tissues.
- 4) Effects of changes in the venous blood volume.

He thought that since there are 4 unknowns, if measurements are made at 5 wavelengths and simultaneous equations with 4 unknowns for the attenuation ratio Φ are set up, accurate SpO_2_ values could be obtained.

**Fig. 5:**
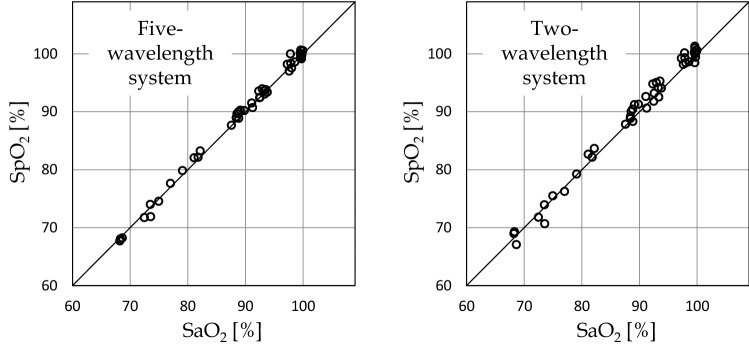
Comparison between the two- and five-wavelength systems

Figure 5 shows an example of a comparison between the two- and five-wavelength systems. Figure 5 (1) shows a comparison with the oxygen saturation measured in blood samples collected at rest, which confirms that the five-wavelength system improves the accuracy. Figure 5 (2) shows a comparison of the SpO_2_ values obtained with the two-wavelength system (b) and five-wavelength system (c) in experiments in which the oxygen saturation was changed by asking the subject to hold the breath and administration of 100% O_2_ by inhalation, while body motion was added (I served as the subject of this study). Probes were attached to both hands, and body motion was added by asking the subject to rub a desk with one hand at random time intervals ranging from 0.5 to 2 s. SpO_2_ values obtained with the five-wavelength system were closer to those on the resting side as compared to the two-wavelength system. As expected at the start of the study, the five-wavelength system was confirmed not only to improve the accuracy, but also the body motion-resistance performance.

### Future issues/prospects

Construction of the theory of pulse oximetry considering the effects of tissues and veins has only been half accomplished. The projects introduced here are still in the research stage, and many problems remain to be resolved before the products are launched in the market. As a researcher working on improving the performance together with Dr. Aoyagi, I would like to follow in his footsteps and try to realize true second-generation pulse oximeters.

Dr. Aoyagi paid attention to pulse wave signals causing noise during measurement of the cardiac output by the dye dilution method and conceived the idea of pulse oximetry. If the theory of pulse oximetry is completed, signals causing noise could also be used as new information in the measurement of arterial oxygen saturation. At present, in perioperative systemic management, cardiac output and great vessel pressures are managed as representative values, and I think that the development of a device that can noninvasively and continuously measure oxygen metabolism at the level of each organ/tissue, which is the ultimate indicator, will contribute to improve the outcome of patients or treatment.

## The development of pulse oximeters in Japan: good competitors, Nihon Kohden and Minolta Camera

Ikuto Yoshiya, M.D., Ph.D.

Honorary Professor, Department of Anesthesiology, Osaka University Medical School. Osaka, Japan.

Akio Yamanishi.

Former Chief, Medical Equipment Division, Minolta Camera Co., Ltd.

(Konica Minolta Co., Ltd., at present), Tokyo, Japan.

The pulse oximeter is currently one of the most indispensable devices in the medical world, like the ECG or the sphygmomanometer. Even in early spring, 2021, as SARS-CoV-2 is still killing thousands of people per day around the world, some local governments in Japan are reportedly providing pulse oximeters to COVID-19 patients who are not hospitalized and have to quarantine themselves at home.

It was indeed a great achievement to invent pulse oximetry, improve its accuracy, and miniaturize the pulse oximeter. All three aspects were essential for the pulse oximeter to become popular worldwide. In April 2020, Takuo Aoyagi, Ph.D., who was the first inventor of the pulse-type oximeter in the world, passed away to the great regret of his colleagues as well as everyone in the medical community who knew of his great achievements. The authors, Akio Yamanishi and Ikuto Yoshiya, and their colleagues, were competitors of Dr. Aoyagi and his team as they worked on the development and clinical application of pulse oximeters.

We dedicate the following brief history of the development of pulse oximetry and its clinical application to our good old rival, the late Dr. Takuo Aoyagi.

### Background

In 1969, members of the research and development group of Minolta Camera watched the astronauts, Neil Armstrong and Buzz Aldrin, become the first humans on the moon. They carried a high-resolution exposure meter, Space Meter 1° aboard their spacecraft, Apollo 11. Earlier, in 1962, a Minolta Himatic Camera with a selenium photocell photosensor was on board the spacecraft, Friendship 7, as Mr. John H. Glenn Jr. orbited the earth. Over the next decade, the photosensor evolved from a selenium photocell, to a cadmium sulfide (CdS) photocell, and then a silicone photodiode (SPD). In 1972, Masaichirou Konishi, who acted as a negotiator with NASA, returned to Japan from the US. On his return, a new research institution was established in Sakai, Osaka, to develop industrial and medical measuring devices. The first medical device developed in the new institution was a photoelectric pulse wave meter (photoplethysmograph). It utilized a light emission diode (LED) and a silicone photodiode (SPD) to generate and analyze transmitted red light through the fingertip. Although the response time of SPD was far shorter than CdS, the device did not become popular in the medical field. This photoelectric pulse wave meter, however, turned out to be the precursor of the fingertip pulse oximeter, OXIMET-Met-1417.

In 1973, Morimasa Takeda et al. reported that the pulsatile wave obtained by the photoelectric measurement of light transmitted through the fingertip represented the fluctuation of the thickness of blood in accordance with pulsation in Japanese journal. This was the moment that the Minolta team became aware of the possibility of developing a non-invasive device to measure arterial oxygen saturation.

The principle of Wood-type ear oximetry (Wood EH and Geraci JE, 1949 [9]) utilized the difference of absorption of transmitted light between when the earlobe was arterialized by warming and when it was made ischemic by pressing it with a translucent balloon. It occurred to Yamanishi and his colleagues that pressing and depressing the earlobe made a kind of pulsation. Having just developed a fingertip pulse-wave meter, they soon created an algorithm to obtain oxygen saturation of arterial blood. In short, the incidental light that travels across the fingertip is absorbed by arterial and venous blood as well as by the tissues of the fingertip (skin, muscle, bone, connective tissue). Assuming that pulsatile fluctuation of the transmitted light is caused solely by the inflow of arterial blood, hemoglobin oxygen saturation can be calculated by the mathematical subtraction of light absorption by fingertip tissues from whole light absorption when arterial blood is full. The Minolta team was more than excited in anticipating an entirely new type of in vivo oximetry, the first since Wood and Geraci [9]. However, a few weeks later, the team members were astonished and disappointed to find a Japanese paper by Aoyagi et al. entitled “The improvement of ear-piece oximeter” in the Proceedings of the 1974 Spring Meeting of the Japanese Society of Medical Electronics and Biological Engineering. Minolta made a patent application as soon as possible, but it turned out the original idea of pulse oximetry by Aoyagi was 1 year ahead of Minolta as was recorded in Aoyagi’s research notes (in Japanese).

Aoyagi’s invention of the principle of pulse oximetry started when he was working on the development of a cardiac output measurement device using the dye dilution method. Aoyagi had noticed that the spectrophotometric dye dilution curve fluctuated with heartbeats. It was a kind of reversed thinking to utilize the pulse wave to estimate arterial oxygen saturation in a non-invasive manner. The authors feel proud of Aoyagi’s great invention originally conceived in Japan. We are also happy to have run a good race with Aoyagi and his colleagues at the same time.

### Development of the fingertip pulse oximeter (OXIMET-Met-1471, Minolta Camera, Co., Ltd.).

After the first patent application made by Minolta for the basic principle of pulse oximetry, Minolta made a more detailed patent application for an apparatus, including circuits, designed by T. Kisanuki and Y. Majima. Calculation of oxygen saturation was made by a simple linear equation from light absorption at the wavelengths 805 nm (isosbestic point) and 650 nm [10].

**Fig. 6:**
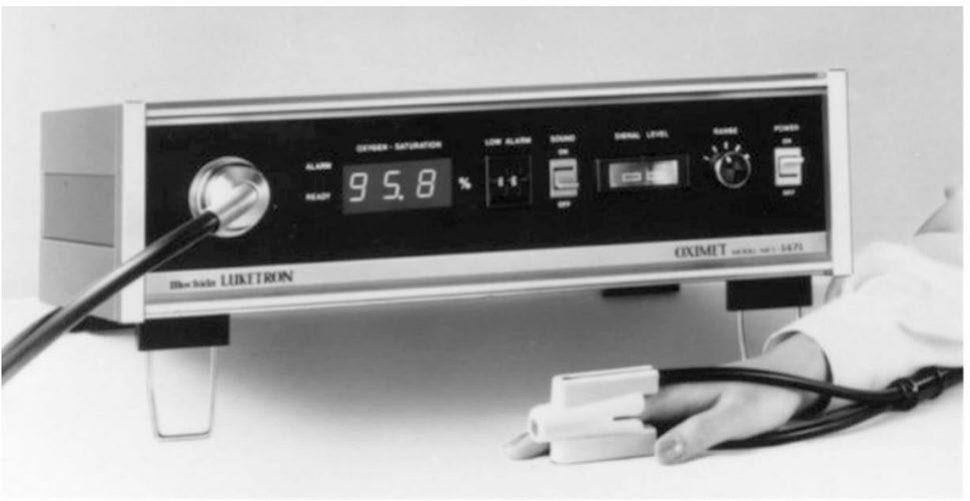
OXIMET Met 1471. The light emitted by a light emission diode travels to the finger probe and the transmitted light is analyzed by a silicon photodiode mounted in the chassis. The incident and transmitted light travels to and from the chassis through the fiberoptics, respectively

Although a light emission diode (LED) was used as the light source for the pulse wave meter, a halogen lamp was employed with the pulse oximeter because of a shortage of LED lights. Because of this, the light emission lamp was incorporated into the chassis of the instrument and incident/emission light traveled through a pair of fiberoptic paths. In 1977, this device was marketed as OXIMET-Met-1471 (Fig. 6).

### Clinical application of OXIMET

In 1977, one of the authors, I. Yoshiya, was consulted by the other author, A. Yamanishi, about the clinical applicability of OXIMET. Yoshiya was then working in the Intensive Care Unit (ICU) of Osaka University Hospital and jumped at the suggestion, because ICU patients needed invasive arterial gas measurements too frequently. OXIMET seemed to have potential as a bed-side monitoring device in critically ill patients.

**Fig. 7:**
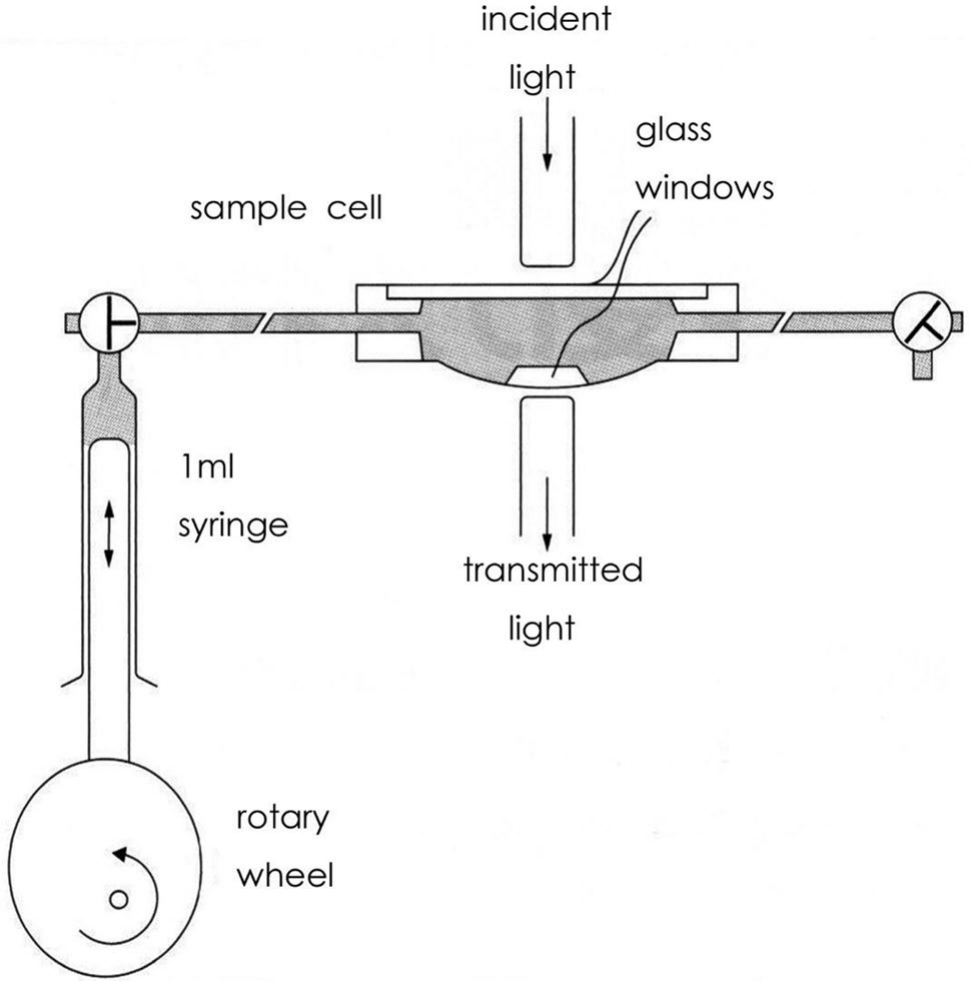
A pulse-generating apparatus for the in vitro pulse oximetry devised by Hamaguri. A rotary pump generates pulse waves in an artificial cell with a pair of translucent glass windows on both sides of the cell. Light emitted by a halogen lamp travels across the windows. The transmitted light is spectrophotometrically analyzed for oxygen saturation by using the pulse oximetry principle. By changing the hematocrit of the sample blood pumped in the cell one can estimate the effect of scattering of light by blood corpuscles. Namely, the oxygen saturation by the in vitro pulse oximetry and that measured by a Radiometer (OSM-2) are compared with different hematocrit of the sample blood

First, we (I. Yoshiya and Y. Shimada, et al.) calibrated the instrument against the oxygen saturation measurements obtained from blood samples of ICU patients [11]. It seemed that the pulse oximeter tended to overestimate arterial oxygen saturation below 90%, compared with the oxygen saturation measured on blood samples with Radiometer OSM-2. The Minolta and Osaka University team tested the hypothesis that the overestimation of oxygen saturation with the pulse oximeter was due to the multiple scattering of incident light by the blood corpuscles. K. Hamaguri (Minolta) devised a model cell to which blood or hemoglobin solution was pumped in and out by a rotary pump (Fig. 7). Using the device, the effect of multiple scattering was successfully compensated for, as was previously reported by Shimada et al. [10]. The accuracy of measurements by OXIMET has been reported by Yoshiya et al. [11] and Sarnquist et al. [12]. The 1980 report by Yoshiya et al. [11] has the honor of being the first publication in English to introduce pulse oximetry as reported by E. C. Jr. Pierce at the 34^th^ E.A. Rovenstine Memorial Lecture. A. Fukunaga (Professor of Anesthesiology, UCLA, Harbor, at that time) informed Yoshiya about this lecture, but regretfully the latter failed to attend the plenary lecture.

The authors utilized OXIMET as a safety monitor in the intensive care ward and in the operating theater. It was also used as a monitor for patients with sleep apnea, but it could not be used routinely due to the size and weight of the finger-probe with fiberoptics.

### Downsizing and weight reduction of pulse oximeters for clinical use

As stated above, the probe and fiberoptic cables of OXIMET were too large and heavy to be used as a bed-side monitor or a monitor for critically ill patients during emergency transportation. It was essential for both the finger probe and the amplifier to be downsized and made lighter to allow the pulse oximeter to be widely used in the clinical field. In 1981, Nellcor and Ohmeda, respectively, released pulse oximeters into the market with small finger probes utilizing LED and SPD. The pulse oximeter soon became popular in the US, particularly in the operation theater. Several years later, the pulse oximeter was imported from the US to its birthplace, Japan.

Minolta also developed PULSOX-7 in 1987 that consisted of a finger probe using LED/SPD and a small, lightweight analyzer with a memory chip. This portable pulse oximeter was widely used for monitoring home oxygen therapy, sleep apnea, and transportation of critically ill patients. In 1990, PULSOX-7 climbed the summit of Mount Xixabangma (height: 8,027 m) carried by Akira Demizu, M.D. who was then a staff anesthesiologist at Osaka University Hospital. Demizu used to be a member of Kyoto University Alpine Club and joined the Kyoto University Medical Research Expedition to Xixabangma in 1990, to investigate the relationship between insomnia and arterial oxygen saturation at high altitude. The successful Himalayan expedition of the pulse oximeter was attained by the ceaseless efforts of the Minolta team to attain size and weight reduction, energy saving, vibration resistance, ease of handling, and cost reduction.

Aoyagi’s continuing effort to develop pulse oximetry was indeed admirable. He led the development team of Nihon Kohden to release OLV-1100/1200 into the market in 1987 and never stopped developing more accurate and motion-resistant pulse oximeters by adopting newer scattering theories and multiple-wavelengths to compensate for the scattering of light by blood corpuscles and body movement of the patient [13]. He gave a speech at the 34^th^ Annual Meeting of the Japan Society of Technology in Anesthesia in commemoration of receiving the 2015 IEEE Medal for Innovations in Healthcare Technology. He said they had successfully settled the problem of body movement. Yamanishi remembers Aoyagi remarked in a chat with him *“You’ll go and find pulse oximeters at supermarkets or electronic shops in the not-too distant future”*. His prophecy has come to pass as of 2019–2021, in the pandemic of COVID-19.

Pulse oximetry was first invented by Aoyagi (Nihon Kohden), but there was competition from Minolta in Japan. The idea soon propagated to the US, where a device was produced using a smaller probe applicable for use in clinical settings. The improved pulse oximeter was imported back to Japan and was further improved and miniaturized. Currently, Konica-Minolta has a pulse oximeter weighing less than 40 g, and pulse oximeters are routinely used on COVID-19 patients worldwide. The authors feel Aoyagi would have been happy about the internationalization of the development of the pulse oximeter.

Finally, we would like to express our sincere condolences to Dr. Aoyagi’s family.

## Aoyagi’s legacy in the US: what influenced its acceptance?

Jeffrey B. Cooper, Ph.D.

Professor of Anesthesia, Harvard Medical School, Department of Anesthesia, Critical Care & Pain Medicine, Massachusetts General Hospital, Boston, MA, USA

Executive Director Emeritus & Senior Fellow, Center for Medical Simulation

While Takuo Aoyagi’s name was not widely known in the US, his invention of a practical way to monitor oxygen saturation has had a huge impact on healthcare, on anesthesiology in particular. He is highly deserving of the honors that have been bestowed on him for this contribution. Now, on his passing, it is fitting that we pay tribute and recognize him for the countless lives his invention has saved and for his creativity and persistence that brought this gift to the world.

I am not an historian. I do not have all the facts or even empirical evidence to claim exactly what influenced the relatively rapid acceptance of this new technology by a profession that typically is skeptical of the latest technology fad and demands evidence before action. In addition, in my experience, anesthesiologists pride themselves on individual responsibility, competence and vigilance to maintain the safety of the care of their patients. Attention to vital signs that could be easily observed, including heart rate, blood pressure, ECG and skin color were thought by many to be sufficient for the attentive anesthesiologist. This is especially so for those who had grown up in a time, where there was little technology and before the term “patient safety” was coined.

How did so many come to accept pulse oximetry in the absence of definitive trials of its effectiveness in preventing harm? Yes, there were some studies to point to its usefulness, but little to justify the expense on a cost/benefit basis. What influences led to almost ubiquitous use of pulse oximetry in just a few years after the introduction of the first practical commercial monitors in the US? I will explore what I think were a few key factors, but without the data I cannot draw definitive conclusions.

I think the most critical factor for accepting pulse oximetry is that it tapped into a visceral desire for all anesthesiologists to eliminate the guesswork to ascertain the state of a patient’s oxygenation. I do not know who first realized the huge potential, but Bill New, an anesthesiologist and engineer certainly played a key role as did his colleague and partner in the business, Mark Yelderman, also an anesthesiologist. From their own clinical experience, they recognized that having such information, updating continuously within a few heart beats, would make any anesthesiologist feel a bit less anxious, especially during critical moments.

**Fig. 8:**
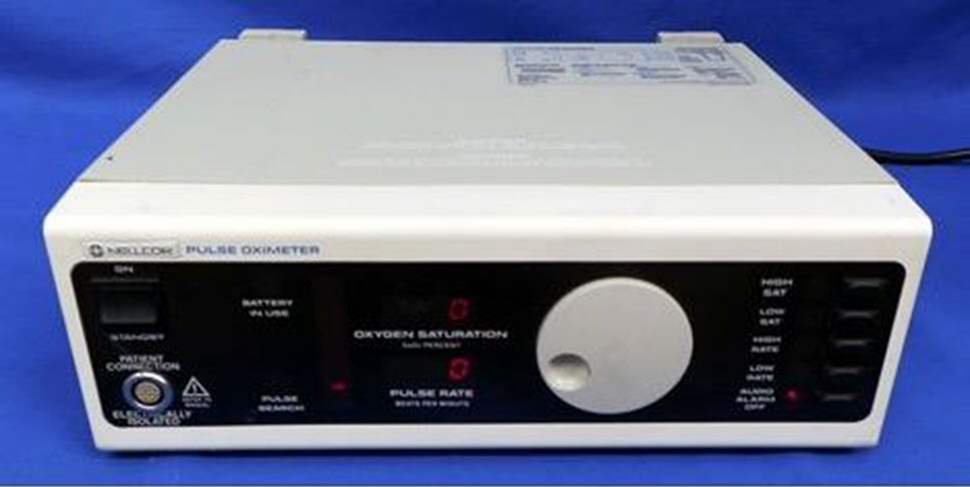
N-100 Pulse Oximeter (1983, Nellcor)

Much credit has to be given to the design of the N-100 Pulse Oximeter (Nellcor), especially its simplicity and brilliant human factors design features (Fig. 8). Getting such critical information from a single number, clearly displayed via LED’s (a relatively new technology at the time), was a game-changer. The ease of application of the sensor also contributed to easy use and acceptance. The look and function of the large adjusting knob were elegant and practical. Perhaps, the most appealing feature was the changing sound of the pulse tone with decreasing saturation. My guess is that the combination of these attributes was the most important reasons for quick adoption in anesthesia. This is a good example of remarkable human factors being heavily responsible for a lifesaving technology, not just the measurement itself.

I suspect many believe that the standards for minimal monitoring during anesthesia established by the American Society of Anesthesiologists (ASA) in October 1986 played a role in dissemination of this innovation. While they may have had some influence on raising awareness of the need for some monitoring technologies, the standards did not at first require pulse oximetry or even strongly encourage its use. The ASA standard was a direct evolution from the standard promulgated by the Anesthesia Departments of Harvard Medical School in 1985; that standard did not mandate pulse oximetry. The rationale in developing a standard (I was there and promoted that rationale) was to first establish the precedent for a basic standard. To achieve acceptance that meant including only items that had stood the test of time and that no reasonable anesthesiologist could argue against. We thus could not immediately require pulse oximetry, because there was still even little widespread personal experience among anesthesiologists. While including a requirement for it would have been prescient, in the absence of data or wide experience, the idea of a standard may have been successfully opposed by vocal skeptics who might have a strong voice in opposition to any standards.

The rationale for the original ASA “Standards for Basic Intra-Operative Monitoring” was similar, as described in an article in the Spring, 1987 APSF Newsletter [14]:

“The ASA committee debated whether to include capnography and pulse oximetry as the “standard of care”. At that time, it was felt impractical to mandate very specific (and very expensive) high tech equipment when the greatest focus of the effort was the general extension of the vigilance of the anesthesiologist. The committee also considered the questions of the consistency of performance of these two instruments and the availability at that time relative to the potential demand. However, E. C. (“Jeep’) Pierce, M.D., committee member and past president of the ASA, now states, “Capnography and oximetry are becoming so widespread that they will be functional standards. Projecting current trends, it is likely that by the end of 1988, enough oximeters will have been sold for there to be one available for every operating room in the country”.

It was not until 1989 that the ASA standard was amended to require pulse oximetry for all anesthetics. It was likely that almost all anesthesiologists were by then using pulse oximetry for every anesthetic.

Over the years, I have heard it said that The Anesthesia Patient Safety Foundation strongly influenced adoption of pulse oximetry. Being one of the founders of APSF, I know that we were careful not to promote specific technologies over others because of concerns of any appearance of conflict of interest, since some of those companies donated to the organization. I do suspect that APSF’s strong advocacy for patient safety and focus of attention on errors in practice raised awareness of all anesthesia providers about the need to take stronger measures than just relying on their own vigilance.

I think that there was another strong force that was responsible for relatively rapid spread of pulse oximetry in anesthesia. Fineberg et al., in 1978, reported on a survey of how new ideas were adopted into practice in anesthesiology using three different examples [15]. While publications are generally the most influential means to persuade anesthesiologists to adopt new ideas into practice, learning from colleagues is a close second influence. Those findings likely applied to the spread of pulse oximetry, which was introduced only a few years after that study. I suspect that it often happened that anesthesiologists heard excited anecdotes from their colleagues about how valuable this addition to their practice was. Perhaps, they heard of a great save resulting from early discovery of an error or of the confidence during intubation on hearing that so recognizable tone indicating the state of saturation. Such word of mouth may have contributed greatly to pulse oximetry “spreading like wildfire” even in the absence of much empirical evidence of its effectiveness.

Regardless of exactly how the idea spread so quickly, all anesthesiologists and, more so, all patients having an anesthetic, are indebted to Takuo Aoyagi for his marvelous ingenuity that has so greatly contributed to the dramatic improvements in anesthesia patient safety.

## Nellcor: Continuous perioperative oximetry comes to North America

David J. Steward MB BS, FRCPC.

Honorary Professor, Department of Anaesthesia and Pharmacology.

University of British Columbia, Canada.

Instruments intended to non-invasively measure the oxygen saturation of arterial blood were introduced as early as 1942 [16]. Very rarely, their potential value as a monitor in anaesthetized patients was evaluated, as, for example, during pediatric tonsillectomy in Montreal in 1951 [17]. A similar instrument was also used in Toronto to monitor anaesthetized infants during the 1950s in the earliest days of cardiac surgery [18]. However, as late as 1982, in North America, the hemoglobin oxygen saturation of patients during anaesthesia was virtually never routinely monitored. Trans-cutaneous oxygen tension tcpO_2_ monitors were developed and marketed in 1976 and soon were in use in NICU. However, these were generally unsatisfactory for use during anaesthesia. Their accuracy was compromised by anaesthesia induced changes in cutaneous blood flow and also by the direct effect of volatile agents on the sensor [19].

The Hewlett-Packard Company marketed an ear oximeter in 1976. This was cumbersome to apply and was never widely adopted. Its performance during anaesthesia in healthy volunteers was reasonably accurate, but the reliability during anaesthesia induced circulatory disturbances was uncertain [20]. There was obviously interest amongst anaesthesiologists in the possibility of monitoring arterial oxygenation, but there was as yet no simple easy way to reliably accomplish this.

**Fig. 9:**
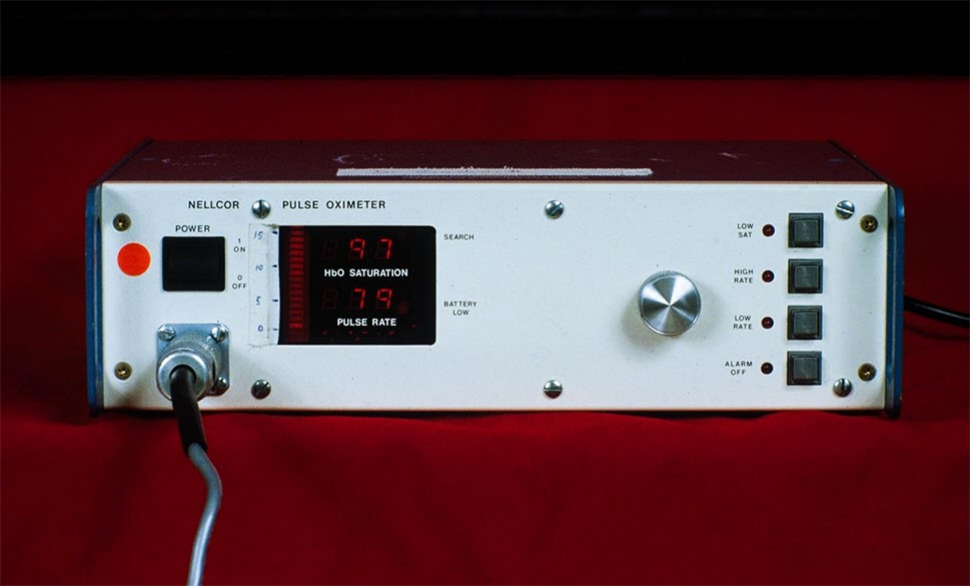
Nellcor Pulse Oximeter Prototype as delivered to us in 1982. Note the absence of the "N-100" designation, this was added at a later date as N-100A. The first commercial model was called N-100B.

In early 1982, when I was serving as the Head of the Department of Anaesthesia at the Hospital for Sick Children (HSC) in Toronto, then the largest academic pediatric service in North America, I had a visit from Dr. William New. He brought with him a prototype of a pulse oximeter (Fig. 9). This machine needed no calibration and was very easy to apply to the patient using a flexible adhesive disposable sensor, or a reusable clip-on sensor. He asked if we would be prepared to evaluate this machine on some of our patients.

At that time (1982), I understood that Dr. New had been an anaesthesiologist at Stanford University and that previously he had been a graduate electrical engineer at Hewlett Packard. He had now become an entrepreneur and was forming a company to produce and market this oximeter. He had a PhD in physiology from UCLA (1963) and an MS in business from Stanford Univ. (1981).

We agreed to conduct a study and I assigned a postgraduate fellow, Dr. Rainer Deckhard to this project. We studied the performance of the prototype in a series of cardiac surgical patients between 6 months and 9 years of age and found good correlation with co-oximeter readings on withdrawn blood samples. We also studied its performance in a series of preterm infants in the NICU who were also being monitored by a tcPO_2_ monitor. Thus, we compared the two methods of monitoring directly. We reported our findings in 1984 in Critical Care Medicine [21]. It is of some interest that the publication of our paper was considerably delayed in the review process. It was finally published unchanged. Much later, I discovered that this delay had been due to the action of a single reviewer, who, it appeared had an interest in a company that marketed tcPO_2_ monitors!

In mid-1984, I left HSC Toronto to assume the position as Chief of Anaesthesia at the newly opened British Columbia's Children's Hospital in Vancouver. When I arrived there, I found that on the proposed budget for the department was a tcPO_2_ monitor! I cancelled this order and replaced it with an order for Nellcor 100 pulse oximeters. By the middle of 1985, we had one Nellcor pulse oximeter in every operating room.

The model N-100 was marketed by Nellcor in 1983 with an extensive advertising campaign. They mass produced a machine which was extremely easy to apply to the patient, required no calibration, and appeared to reliably follow arterial oxygen saturation levels. Although Nellcor’s promotions through ASA and IARS meetings and in the journals were outstanding, it was a product that sold itself and it was a product whose time had come!

Sales of the machines rapidly accelerated, and though there were some other models available, "Nellcor" and "Pulse Oximeter" became almost synonymous. By the end of 1986, a pulse oximeter was in nearly every operating room, recovery room, and intensive care unit in North America. The Anesthesia Patient Safety Foundation bulletin of Spring 1987 predicted:

"Projecting current trends, it is likely that by the end of 1988, enough oximeters will have been sold for there to be one available for every operating room in the country”.

In October of 1986, the American Society of Anesthesiologists recommended that every anesthetized patient should have arterial oxygen level constantly monitored.

“During all anesthetics, a quantitative method of assessing oxygenation such as pulse oximetry shall be employed. When the pulse oximeter is utilized, the variable pitch pulse tone and the low threshold alarm shall be audible to the anesthesiologist or the anesthesia care team personnel [22].”

In 5 years, something that had almost never been done before became a recommended standard of practice. An original Nellcor N-100 monitor is on display in the Wood Library-Museum of Anesthesiology in Chicago. The accompanying catalogue description includes the following:

"Like most modern medical devices pulse oximeters represent advancements in technology and a culmination of years of knowledge in basic and clinical sciences gained by the trial and error of numerous researchers. Japanese researcher, Takuo Aoyagi is often recognized for putting the "pulse" in pulse oximetry by using the waveform produced by the arterial pulse to measure and calculate SpO_2_. He first reported on the work of his team in 1974."

## Pulse oximeter: dissemination in neonates and its history

Hiroshi Nishida, M.D., Ph.D.

Professor Emeritus, Tokyo Women’s Medical University, Tokyo, Japan.

### Introduction

Experts have already discussed the basic principles and history of development of pulse oximeters in other sections. Therefore, in this chapter, I provide an overview of the history of introduction of pulse oximeters in neonatal care in Japan. I also explain the significance of development of blood oxygen level monitors dedicated to neonates, particularly premature infants, in the context of their clinical significance in reducing the risk of retinopathy of prematurity and hypoxic brain damage.

### Characteristics of medical care of neonates, particularly premature infants

Human babies, even full-term (40 wks) infants, are born with physiological immaturity, in compensation for the large brain supporting the high level of human intelligence. This can be easily imagined from the fact that while other large mammals begin to walk independently almost immediately after birth, human neonates can just begin to hold their heads up by 3 months after birth and begin to walk alone by around the age of 1 year. In particular, in regard to respiration, gas exchange between the fetus and the maternal placenta occurs through the umbilical cord and fetuses make breathing-like movements in the womb, but pulmonary function only begins to be established at birth. Infants easily become apneic due to pulmonary immaturity, and sudden infant death syndrome (SIDS), which occurs due to delayed recovery from sleep apnea, is a well-known entity.

For this reason, in the history of medical care of premature infants, apnea monitors are counted as the medical devices that have contributed the most to preventing SIDS and brain damage due to hypoxia. In 1972, when I became a neonatologist, I remember that young residents, equivalent to human monitors, used to be stationed near the incubators for premature infants all day long; at present, however, the world of medical care has changed dramatically, and premature infants are monitored by pulse oximetry in every NICU.

### Retinopathy of prematurity and blood oxygen levels

Retinopathy of prematurity (ROP) is thought to be caused by the toxicity exerted by high concentrations of oxygen. At one time in the US, instituting the limitation of the oxygen concentration that could be administered to premature infants to 40% decreased the incidence of ROP, but increased the frequency of brain damage occurrence, which triggered a discussion on whether it was more important to protect the eyes or the brain. Since, it has been revealed that ROP is caused not by high oxygen concentrations per se, but by retinal tissue ischemia induced by constriction of the retinal arteries caused by oxygen supplementation at high concentrations, which has led to a focus on the necessity of adequate measurement of the arterial blood oxygen levels. However, the partial pressure of oxygen in arterial blood, specimens of which are difficult to obtain, was not found to be correlated with the incidence of ROP. This suggests that an accurate assessment cannot be made by a single measured value of the partial pressure of oxygen, which changes continuously, but by continuous monitoring of the arterial oxygen tension. Namely, it became clear that devices that allow continuous, not intermittent, monitoring and recording of the arterial blood oxygen levels are required to prevent ROP, which has led to the development of transcutaneous partial pressure of oxygen monitors and pulse oximeters.

Widespread use of these medical devices has greatly decreased the incidence of acquired blindness in premature infants. However, at present, while many very preterm infants with a birth weight of 500 g or less (approximately 23 weeks of gestation) grow normally, ROP is encountered even in infants managed with adequate blood oxygen levels. Therefore, immaturity is at the root of the pathogenesis, and I would like to add that further studies are ongoing.

### From transcutaneous partial pressure of oxygen monitors to pulse oximeters

Transcutaneous partial pressure of oxygen monitors were initially developed to continuously monitor the blood oxygen levels in neonatal care, and there is the late Dr. Itsuro Yamanouchi’s interesting episode of their introduction in Japan. He believed that noninvasive care is important in the medical care of premature infants and developed a transcutaneous bilirubin measurement method, which has also spread from Japan to the world, together with Akio Yamanishi of Minolta Co., Ltd (who also invented the fingertip pulse oximeter, the currently commonly used type of pulse oximeter around the world).

In transcutaneous partial pressure of oxygen measurement, the skin is warmed to arterialize the capillaries and the blood partial pressure of oxygen is measured using the principle of polarography. At first, a German obstetrician, Prof. Huch, developed this device to measure fetal scalp partial pressure of oxygen, and it was Dr. Itsuro Yamanouchi who demonstrated that the device is also clinically useful for neonates, which led to its commercialization by German and Swiss companies. Thus, in contrast to pulse oximeters, transcutaneous partial pressure of oxygen monitors were developed in Europe, while their usefulness in neonates was demonstrated by Dr. Yamanouchi in Japan. However, the sensors of transcutaneous partial pressure of oxygen monitors must be replaced every 2–3 h, because they could get hot (heated up to 43.5 °C), and when the skin of the premature infants begins to thicken with age, the accuracy of the measurement would decrease; therefore, transcutaneous partial pressure of oxygen monitors began to be replaced by pulse oximeters of oxygen saturation monitoring. At that time, Dr. Yamanouchi was concerned that the use of pulse oximeters in neonates might be associated with a gradual increase in the incidence of ROP, because in the measurement of high blood oxygen levels, a slight difference in the arterial oxygen saturation corresponds to a large difference in the arterial partial pressure of oxygen [23]. Fortunately, however, the incidence of ROP did not increase with the introduction of pulse oximeters, due to the improvement in the accuracy of pulse oximetry measurements and the careful management by nurses and other staff working in NICUs across Japan.

### History of appropriate dissemination of pulse oximeters in Japanese neonatal care

As mentioned in other chapters, the principle of pulse oximetry was discovered in Japan, but Japan lagged far behind the US in allowing its clinical application to spread as a monitor. In neonates, in particular, this is partly because transcutaneous partial pressure of oxygen monitors had already been widely introduced, and furthermore, because not only medical device manufacturers, but also medical professionals did not yet fully understand the usefulness of pulse oximeters in neonatal care.

In those days, pulse oximeters were very unpopular; physicians and nurses working in Japanese NICUs felt that pulse oximeters were useless, because they were too sensitive to body motions and generated too many false alarms, and that they were unreliable as compared to transcutaneous oxygen monitors. In addition, the manufacturers themselves also had insufficient knowledge, and I remember once when I complained that I was having trouble with the breakage of the wires of the sensors attached to the hands and legs of the neonates, the electrical engineers who visited the NICU in response to my complaint, who had previously exclusively observed only anesthetized, immobilized adult patients, were astonished to observe the continuous body motions of the neonates in incubators. Furthermore, low temperature burns due to pulse oximeters, which they had bluntly said were unbelievable, were considered to result from local heat retention caused by slight wire breakage in the light-emitting part and inhibition of perfusion due to compression. Subsequently, various technical improvements have been made, and pulse oximeters have evolved to sufficiently withstand daily use in NICUs; however, the experiences with early models left a strong impression on the users, which could explain why the use of pulse oximeters has spread rather slowly in Japanese neonatal care.

Dr. Katsuyuki Miyasaka took up the mission to remove this unfavorable impression and disseminate the clinical importance of pulse oximeters in NICUs throughout Japan. Dr. Miyasaka organized the “Hakone workshop on neonatal pulse oximeters” at the Fujiya Hotel, Miyanoshita, Hakone, which is famous as one of the three major classic Japanese hotels, on July 11, 1987, to which he invited the chiefs of major NICUs across Japan. The original purpose of the workshop was to determine whether the algorithms of foreign pulse oximeter manufacturers for calculating the arterial oxygen saturation were also applicable to Japanese neonates, by comparing pulse oximeter (Omeda Biox 3700) measurements with measurements of the arterial oxygen levels made in samples collected via an arterial line, as part of the research projects of the Psychosomatic Disorder Research Neonatal Management Group of the Ministry of Health and Welfare (leader: Kazuo Okuyama, co-investigator: Hiroshi Nishida). Another purpose was to have Dr. Joyce Peabody, a neonatologist from Loma Linda University, provide a thorough explanation about the principles of blood oxygen monitors and discuss the actual status of use of pulse oximeters in NICUs in the US, so that Japanese neonatologists could clearly understand the principles and show greater interest in the use of pulse oximeters. At that time, Dr. Miyasaka mentioned differences between functional oxygen saturation (So_2_) and fractional So_2_, from the point of view of neonatal care: methemoglobin levels increase when nitric oxide (NO) inhalation therapy is provided for persistent pulmonary hypertension of the neonate (PPHN) and the carbon monoxide (CO)–hemoglobin concentration increases with bilirubin production.

This, along with advances in the functioning of pulse oximeters, such as improvements of the measurement accuracy and simplicity of use, led to improvements in the reliability of pulse oximeters. As a result, pulse oximeters are currently used routinely to monitor the blood oxygen levels even in neonates.

### Concluding remarks

In the chapter entitled “Japan’s contribution to the world in the development of blood oxygen monitors that protect the brain and eyes” in my book entitled “Trajectory of the Development of Japanese Neonatal Care (in Japanese), Medicus Shuppan Publishers, Co., Ltd., 2015,” I stated that *“the historic achievements of Dr. Itsuro Yamanouchi in demonstrating the efficacy of transcutaneous partial pressure of oxygen monitors in neonates and of Dr. Takuo Aoyagi in discovering the principles of pulse oximetry, are the pride of Japan, worthy of special mention, as they have contributed to saving the lives of many neonates and preventing blindness due to ROP worldwide.”*

In particular, pulse oximeters are used for medical purposes in wide areas not limited to neonatal care, and Lindahl from Sweden, who was a member of the Nobel Committee and gave a keynote speech at the 1997 Annual Meeting of the Japanese Society of Pediatric Anesthesiology stated that Dr. Aoyagi’s contribution to humanity deserves a Nobel Prize. Dr. Aoyagi stated, in his letter to me (September 2017), that he was trying to obtain theoretical pulse oximetry values based on the theory of scattered light to leave his footprints as a researcher deserving it. He accomplished this task before his demise, and his achievements went down in history, although he missed the Nobel Prize.

## How pulse oximetry influenced medicine and how its evolution will influence medicine

Joe Kiani, BSEE, MSEE.

Founder, Masimo & Patient Safety Movement Foundation, Irvine, CA, USA.

In 1974, Japanese bioengineer Dr. Takuo Aoyagi made one of the most impactful inventions in patient monitoring and with it improved patient safety [24]. Dr. Aoyagi was in pursuit of noninvasive cardiac output monitoring, but instead invented pulse oximetry. Prior to Dr. Aoyagi’s invention, oxygen saturation monitoring was relegated to the laboratory due to the bulkiness of the devices that measured it, such as the 8-wavelength ear oximeter developed by Hewlett Packard. Dr. Aoyagi filtered the arterial pulse signal and normalized it against all of the variables that the 8-wavelength ear oximeter had to account for (such as tissue thickness, skin pigmentation and other factors) by dividing the filtered arterial pulse information by all the information the photo detector measured [25]. This was Dr. Aoyagi’s so-called “AC/DC” invention. Then, dividing the ratio at one wavelength (sensitive to oxygenated hemoglobin) by the ratio at another wavelength (sensitive to both oxygenated and deoxygenated hemoglobin) gave us the “ratio of ratios,” which is correlated with arterial oxygen saturation (empirically derived from laboratory studies). Through his pioneering work and meticulous research, Dr. Aoyagi invented conventional pulse oximetry. Today, many consider pulse oximetry to be the “fifth vital sign.”

Dr. Aoyagi’s invention of pulse oximetry was a significant advancement in patient monitoring and safety. Before the advent of pulse oximetry, the rates of anesthesia-related deaths and brain injury were very high, because the only methods available to the anesthesiologist to check arterial oxygen saturation were intermittent blood lab tests or observing the color of the lips, purple indicating low oxygen saturation. Although a 20,000-patient study showed no difference in mortality of patients with or without pulse oximetry [26], anesthesia-related fatalities dropped from 1-in-10,000 to 1-in-1 million after the advent of pulse oximetry [27]. Clinicians immediately recognized the immense value of pulse oximetry and it quickly became a standard of care in operating rooms and intensive care units.

Dr. Aoyagi’s pulse oximeter paved the way for Masimo’s invention of Measure-through-Motion and Low Perfusion technology [28]. Continued innovation is paramount to improving patient outcomes. While Dr. Aoyagi’s invention assumed all blood that pulsates is arterial blood, Masimo’s invention accounted for venous blood during motion by separating venous blood pulsation from arterial blood, hence providing increased accuracy for specific patients, such as the poorly perfused in the OR, ICU patients, such as neonates, and awake patients in post-surgical wards and at home.

Multiple clinical studies using the Measure-through-Motion technology have demonstrated a host of successful patient outcomes, including a dramatic reduction in Retinopathy of Prematurity (ROP) in NICUs [29], the ability to detect Critical Congenital Heart Disease (CCHD) [30], and to detect patient deterioration from Opioid Induced Respiratory Depression (OIRD) [31, 32]. Pulse oximetry is no longer limited to the hospital. Clinicians can now monitor their post-surgical patients that have been prescribed opioids or COVID-19 patients who may not need ICU care in the comfort of their homes.

In the future, pulse oximetry may indeed become the most important of the five vital signs. Not only because oxygen is necessary for life, but its measurement and principles of measurement have resulted in a greater understanding of the heart, lungs, and systemic issues. Today, the modern pulse oximeter, which we call the Pulse CO-Oximeter, has the capability to measure not just SpO_2_, but pulse rate, respiratory rate, fluid levels, respiration effort, total hemoglobin, carboxyhemoglobin, methemoglobin, perfusion index (PI), and oxygen reserve index (ORi).

Takuo Aoyagi’s curious fascination and ardent research gave the world the gift of pulse oximetry. He had an idea and he made it his life’s work to realize it. In the years before he passed away, he even brought out his noninvasive cardiac output monitor. Clinicians, patients and engineers owe Dr. Aoyagi a debt of gratitude. We will remember Dr. Aoyagi for his boundless work, significant contributions to clinical care, and his kindness and humility.

## Pulse oximetry and innovation—ability to translate invention to innovation

Hirokazu Ogino, MBA, MS,

President and Chief Executive Officer of Nihon Kohden Corporation, Tokyo, Japan.

**Fig. 10:**
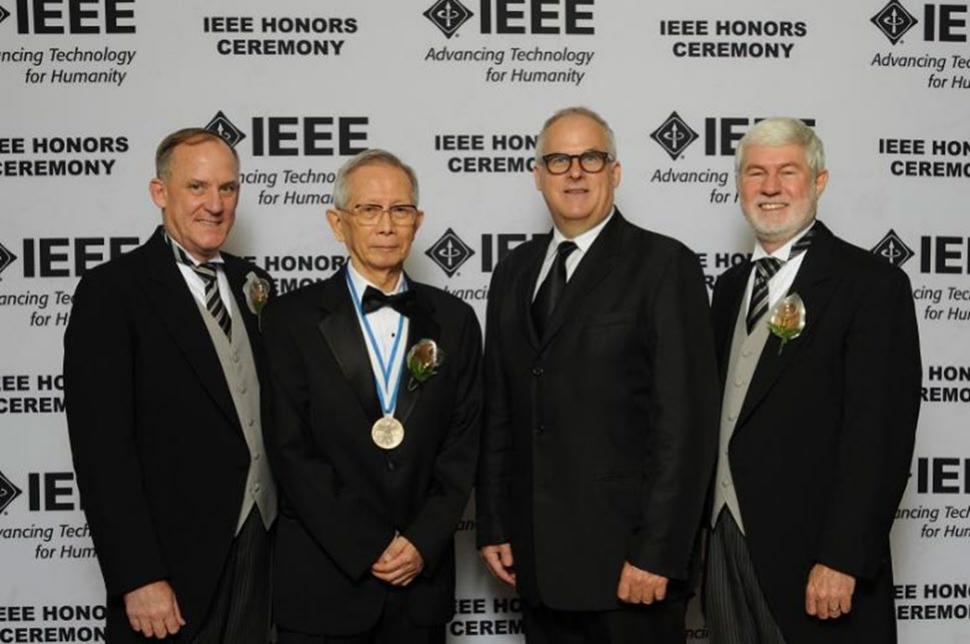
A souvenir photo from the 2015 IEEE Honors Ceremony

On June 20, 2015, I witnessed a historical moment in the ballroom of the Waldorf Astoria hotel in New York. Dr. Takuo Aoyagi, who invented pulse oximetry, was being awarded a Medal for Innovations in Healthcare Technology, the first for a Japanese, by the Institute of Electrical and Electronics Engineers (IEEE), the world’s largest association for electrical and electronic engineers, the founders of which included greats like Thomas Alva Edison. Even though he was 79 years at that time, Dr. Aoyagi walked resolutely on to the stage in a tuxedo, gave an acceptance speech which, even if not fluent, was clear and powerful, and received a great ovation [33]. This was a moment of recognition of the great invention by Dr. Aoyagi and of the contribution of Japan to medical safety worldwide (Fig. 10).

Nihon Kohden Corporation also takes great pride in the invention of pulse oximetry by Dr. Aoyagi, although it has since become a story of success and failure. Innovation is said to represent invention translated into implementation. Our company successfully invented pulse oximetry, but has fallen behind other companies in its implementation.

**Fig. 11:**
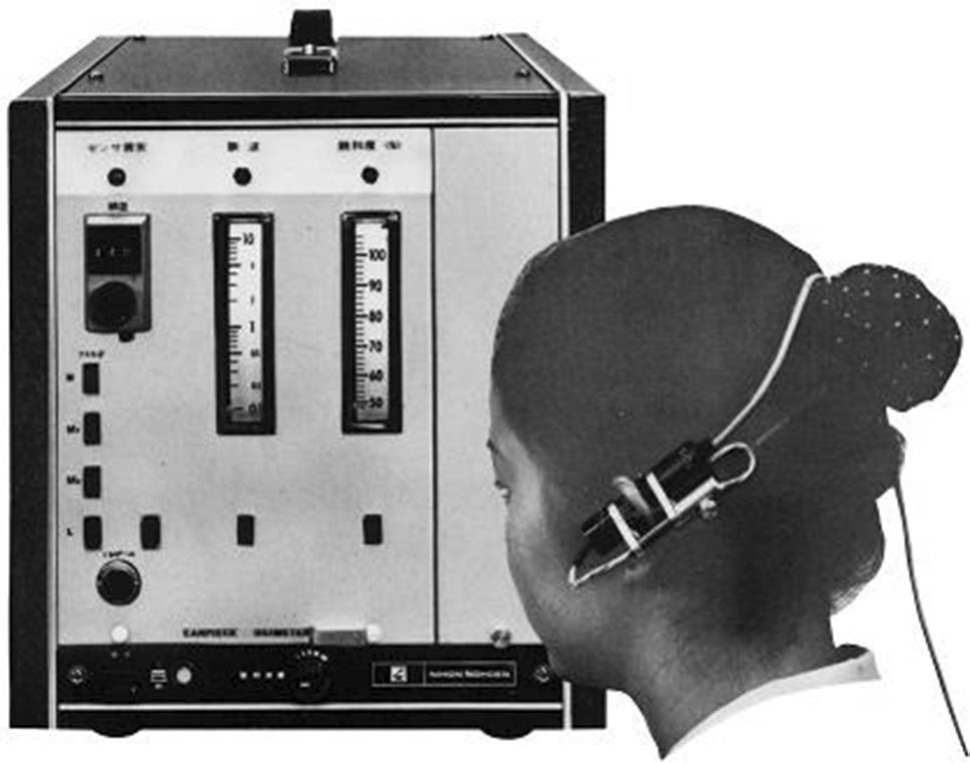
The world’s first pulse oximeter (ear oximeter OLV-5100)

Looking back into history, pulse oximetry may have been invented a bit too early, like many other inventions. In 1974, when Dr. Aoyagi invented the principles of pulse oximetry, semiconductor devices, such as light-emitting diodes (LEDs) [34], had not yet become well established, and small probes suitable for clinical use could not be developed (Fig. 11). Therefore, it took nearly another 10 years before LED-mounted small probes were introduced. In addition, the market for continuous monitoring of blood oxygen saturation was still not recognized. A change occurred in the first half of the 1980s, when intraoperative medical accidents became an issue in the US. Nellcor, Inc., which was founded by Dr. New, caught the wave of technological innovation and market changes at this time, and paved the way for the implementation of continuous oxygen saturation monitoring. In the 1990s, Masimo Corporation, founded by Kiani, distinguished itself by creating the current market for pulse oximeters.

Why did Nihon Kohden Corporation fail to seize the market opportunity, even though it invented the principles of pulse oximetry? A look into this problem revealed the deep gap between invention and implementation. What is required to overcome this gap is an ability different from inventive ability alone; that is, the ability to disseminate innovative technology to the market, in other words, marketing capability. Regarding the contribution of these companies to the development of pulse oximeters, Dr. Aoyagi stated the following in the Japanese journal, in his contribution in 1990:

“Nellcor, Inc. (snip) widely advertised the clinical importance of pulse oximeters.”

Using this past failure as a lesson, Nihon Kohden has since attempted to disseminate new inventions derived from pulse oximeters worldwide. Estimated continuous cardiac output (esCCO), first invented by Dr. Yoshihiro Sugo of our company, is a non-invasive measure of the pulse wave transit time from the electrocardiographic waveform and the pulse wave from a fingertip pulse oximeter, and provides a continuous estimate of the cardiac output [35–37]. Considering that Dr. Aoyagi conceived of the principles of pulse oximetry during the study of cardiac output measurement using the dye-dilution method, I am deeply moved by the fact that the cardiac output can also be estimated by a different method using pulse oximetry.

**Fig. 12:**
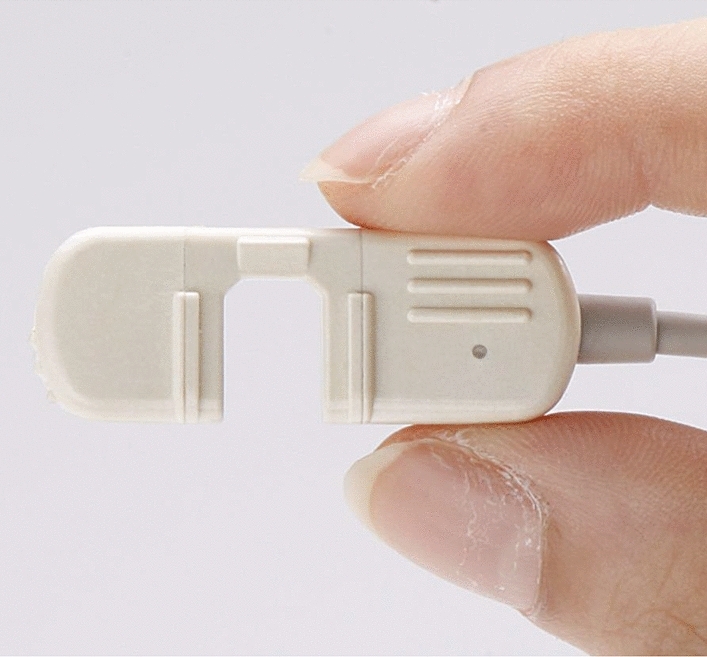
Mainstream CO2 sensor cap-ONE (TG-980P)

**Fig. 13:**
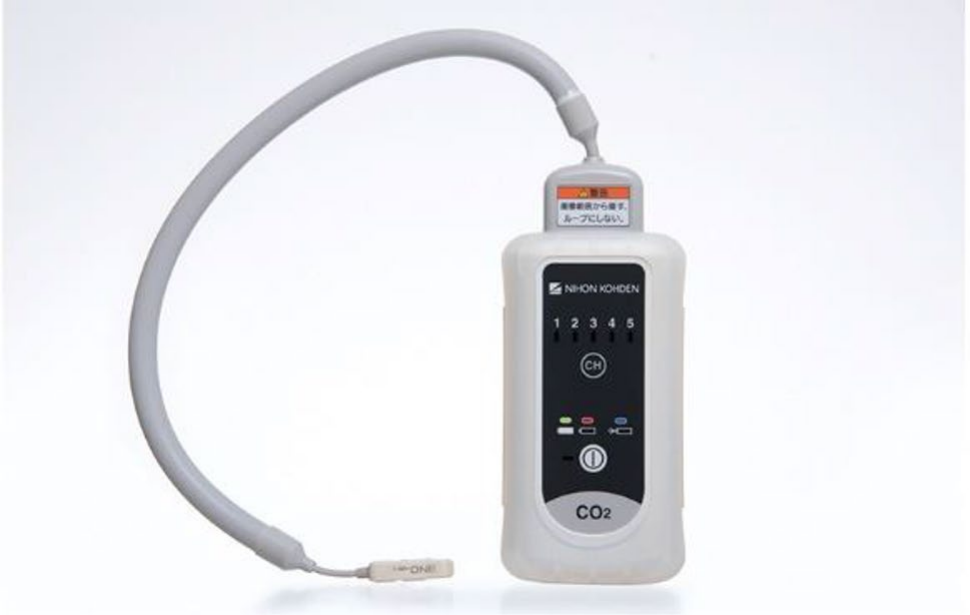
MRI-compatible CO2 sensor module (TG-MR9T)

Similarly, “cap-ONE,” which is the world’s smallest and lightest mainstream CO_2_ sensor developed by Dr. Shinji Yamamori of our company, is more like a breakthrough toward implementation than invention. The achievement of microminiaturization (approximately 4 g, one-fifth compared with the previous model) using an original technology enabled its application into various clinical settings (Fig. 12). Recently, our company successfully implemented magnetic resonance imaging (MRI)-compatible mainstream CO_2_ monitoring for the first time in the world (Fig. 13) [38–40].

To develop the ability to translate invention to innovation, this is the challenge that Dr. Aoyagi and the history of pulse oximetry left us.

## The role played by an import company in introducing pulse oximetry to Japan—Me, my company and pulse oximeters

Yasuhiko Sata.

President and Chair, Tokibo, Inc., Tokyo Japan.

### Introduction

The pulse oximeter was invented by Dr. Takuo Aoyagi of the company Nihon Kohden. The device originated in Japan, but at that time (late 1970s to mid-1980s), the device was hardly sold in Japan at all. Our company works closely with both Japanese and foreign anesthesiologists in selling ventilators and anesthetic devices, so we were keeping a close eye on the developments in the market for pulse oximeters in the US. Perhaps, because Japanese clinicians were not involved in the development of pulse oximetry, the devices from Japan lacked in both function and design. Understanding of and interest in pulse oximeters by Japanese anesthesiologists was not very high.

### How I came upon my first pulse oximeter

While pulse oximeters that use sensors attached to the fingertips or ears to measure oxygen saturation in the blood in a non-invasive manner are as essential as echocardiograms and respiratory monitors in modern medicine, this was not the case in Japan around 1983. Very few physicians paid attention to this new technology. The US is a free country that gives all people a chance to start their own venture companies and many clinicians have started such companies. Nellcor is a venture company started by a clinical anesthesiologist (with a background in electrical engineering), Dr. New, to manufacture and sell pulse oximeters. He designed and sold a small, but sophisticated device and gathered attention from many anesthesia departments in the US.

Our company approached Nellcor around 1983 after realizing the importance of pulse oximetry, despite the lack of interest in Japan. The negotiations started out well, but as they entered the final stages towards signing a contract, Nellcor told me to meet with the vice-president of a large automobile parts manufacturer (SE, Inc.). I was told that I would understand why the meeting was important when I went. The moment I walked in the door, the vice-president said *“Don’t you know that our company manufactures the diodes for Nellcor’s pulse oximeter, one of its most crucial parts. Our SE Company has the rights to sell this product in Japan.”* I was hit with this one-sided announcement without warning. I thought, *“What were all those negotiations about anyway? This is ridiculous!”* I felt like a fool and had great regret.

The work of a retail company selling medical devices is not just to simply sell machines. It is important to have background knowledge of, experience with, and technical knowledge of the product to provide the best service and advice. This will result in the development of a good relationship with the customer. I was disappointed by Nellcor’s decision to allow SE Company to sell their products, even though they were not familiar with the way things worked in the medical industry in Japan. I did not think that it was in Nellcor’s best interest.

### Introducing Ohmeda’s pulse oximeter and our sales activity

About a year later, the US journal “Anesthesiology” published a review of the pulse oximeter developed by Ohmeda BOC Healthcare. Ohmeda’s product (Ohmeda Biox 3700) was found not to be inferior in accuracy compared to Nellcor’s device [41].

I still had bad feelings toward Nellcor and SE Company, so I contacted the president of Ohmeda Japan right away and started to negotiate the right to sell their product. Things did not go well at first, but eventually, we convinced them that ours was the only company with the experience needed to sell a new product like this. We finally signed a contract as their sales representative. We started a drive to sell this product. It was not well known in Japan and in order for the Biox pulse oximeter to make inroads in Japan, it was important to deal with the issue that it was not covered by Japanese health insurance. We also had to negotiate with Ohmeda to reduce their price so that Japanese hospitals could afford to buy them. We cut the price in half, from 3,600,000 yen to about 1,800,000 yen. We were encouraged by Japanese anesthesiologists who predicted that one day every patient would have their own pulse oximeter.

We also felt that it was important to improve the product to make it more appealing to anesthesiologists. Pulse oximeters produce stable readings of 97 to 98% in healthy people, and unless the person is very sick, it will not show lower readings. Using this to our advantage, at the suggestion of an anesthesiologist, we talked to Ohmeda about adding a brand-new function, the first in the world, of showing the waveform so you could see the validity of the measurement at one glance. Ohmeda implemented this immediately. The device started selling very well due to its low price and the visual functionality of its continuous waveform display (showing the normal status of the patient and the device) which increased the confidence medical staff had in the device.

### Broad clinical studies to be covered by national health insurance

It was important to study the clinical efficacy of pulse oximeters as that is what was used to determine whether it would be covered by national health insurance, so following the enthusiasm of physicians at the National Children’s Hospital, the ‘Pulse Oximeter Research Group’ was established and physicians in major hospitals from Sapporo in the north and Okinawa in the south participated in this research. The group came up with a protocol and began clinical studies. Most people with influence in academics in Japan at the time were from public universities and staff physicians who were required to comply with a high standard of conflict of interest guidelines. Thus, the research group formed voluntarily by qualified anesthesiologists was very welcome.

The Research Group, consisting of physicians from participating hospitals, met about once every 3 months for 2 years on a voluntary basis and discussed the clinical use of the device and other issues, presenting a wide range of studies in academic conferences related to pulse oximetry use.　In 1987, Ohmeda held a workshop in Hakone, Japan, the first of its kind in the world outside the field of anesthesia, on how to conduct research on pulse oximeters in newborn infants. We co-sponsored the meeting. The first book in Japanese on pulse oximetry grew out of this meeting. This meeting was the neonatal equivalent of the International Chartridge Meeting held in 1985, outside of London. It was organized by Drs. Payne and Severinghaus, and the naming of SpO_2_ was agreed upon there.

Thanks to the great effort and time spent on research and participation in the research group, even though it took a while, pulse oximetry achieved formal recognition in the national health care system. At last pulse oximetry found a place in the Japanese medical system. The Pulse Oximeter Research Group decided to expand its role after achieving its first purpose. It developed into the Japanese Association of Clinical Monitoring.

### Importance of sale activity characterized to Japanese medical situation

Due to these efforts, Ohmeda’s pulse oximeter was selling well. Nellcor’s device did not sell well, and while it was rumored that SE Company had given up, after a while CM Company, the medical device subsidiary that imported medical devices for a large trading company (IC Company) took over sales. At the time, Ohmeda had the largest share of the pulse oximeter market in Japan. While Nellcor held the largest share in other countries, the reverse trend in Japan was unusual from the point of view of both sides.

Our company has had one mission from the start, and that is not just to sell devices, but to help deepen the understanding physicians and nurses who use these devices have. This applies to selling products from Bird or Newport ventilators. This is invaluable in Japan, where continuing education for post-graduates is not well developed. I think that this mission for our company helps explain why were able to successfully introduce pulse oximetry to Japan and contribute to a healthy spread of its use.

### Termination of the contract and the end of our dealing with pulse oximeters

We thus expanded sales of Ohmeda’s pulse oximeter and were planning on strengthening the market even more. However, suddenly 1 day, Ohmeda told us that they were cancelling our sales contract with them. A new company (OS Company) was formed with Ohmeda and a subsidiary of a large electronic manufacturing company (N Company) called S Company that manufactured and sold medical devices. They planned on selling all over Japan. At the time, the Ohmeda Company itself was caught in the currents of change of the medical setting, which continues even today, where mergers are based simply on monetary relations and not the convenience of customers. Once more it was our fate to accept the loss of our retail stores and the products in which we invested so heavily. Looking back, OS Company’s plans failed completely, and I have heard that it took years to resolve all the issues.

The next step we took was to get the rights to sell the pulse oximeter from the American company, CS (a company that sells products for the ICU). Compared to the previous negotiations, the contract was concluded relatively smoothly. However, at the time change was coming to the market for pulse oximeters. This was the rise of disposable sensors. CM Company, a subsidiary of a large trading company, the new representative of Nellcor in Japan, aggressively tried to attain the number one share of the market. Our company had been left out by the Ohmeda Company, giving Nellcor a better chance. They lent their devices out for free and tried to increase their sales by making money out of selling disposable sensors.

However, CS Company, that dealt with the American market, was too late in putting out their line of disposable sensors. The functionality of the device was not that good, and sales lagged. We considered ending our involvement with the products of CS Company, but we could not find a replacement sales company. To keep our responsibility to the hospitals who had already bought these products, we continued to sell their products in a non-exclusive relationship.

### Topics associated with disposable sensors

It was under these circumstances that we signed a sales contract with DM Company, a subsidiary of a large American airport surveillance device company. This company had disposable sensors that were compatible with the Nellcor device, and they sold pulse oximeters as well all over the world. They also handled devices for veterinary devices. The owner was from India and had close contact with a company that primarily sold X-ray machines to inspect airport luggage. The company was quite successful. We were not sure whether to get involved, as we had never dealt incompatible or generic products, only original products. We were reassured by the size of the company and by their claim that the patent for disposable sensors by Nellcor had expired and they were free to make and sell copies.

Before signing a contract, we too looked into the patent issue and signed a contract based on DM Company’s promise to guarantee the product. We began to sell pulse oximeters and sensors. I felt that our company was better equipped to handle sales in Japan than Nellcor (partnering with the CM Company to sell in Japan). We proceeded to promote these products to the network of people we knew in the ICU, NICU, and Emergency Medicine, who were using our company’s original Newport ventilator. There were some problems with the device and with the sensors, but we solved them through our sales force and technical skills. We were making inroads all over Japan, especially around Nagoya, where we received a very large order.

Nellcor (CM Company) was beginning to show results, and with the purpose of holding onto their customers, started changing the connectors between the device and the disposable sensors so as to make generic products incompatible with it. However, we felt that there were enough older machines around, and enough hospitals that would continue to use the original product, that our business would be all right. Just as we thought the products from DM Company were doing well in the market, we got a registered letter from Nellcor saying that DM was infringing on its patent for disposable sensors.

We gathered a team of company executives and company workers involved in this project and along with a legal team from Japan came up with a strategy. We were in contact with DM by email and telephone, but we were unable to get a clear response from them regarding the patent. We went straight to DM in the US through our American subsidiary, TKB-I. We argued with the president, but were discouraged by his lack of sincerity. In the end, we terminated our contract with DM. Our company has not dealt with pulse oximeters since then.

### Pride of our agency

Our company trades mainly in sales of imported medical devices. We place great importance on the promotion and proliferation of imported clinical devices that are at the cutting edge of the field that might otherwise not be introduced into the Japanese medical field. This is true of the pulse oximeter, as well as the first hemodialysis machines, mass spectrometers, Holter ECGs, etc. We have a policy not to deal with medical devices that are easily accessible in the Japanese market or in a price war. The essence of our business model is to contribute to the health of people through medical professionals, but it is not necessarily an easy way to be profitable. The road is rough, with many barriers, but we are proud of our company.

### Afterword

I am grateful for being given the chance to talk about our experience with spreading knowledge of imported medical devices in Japan and our efforts to increase anesthesia safety through this special memorial to the great achievements of Dr. Takuo Aoyagi, inventor of pulse oximetry. I hope this will be of some use to anesthesiologists in Japan and to people who work in the medical field. It would please me greatly if this report is useful in the field.

## Commemoration of Dr. Takuo Aoyagi’s Impact—a tree that was heard to fall

Robert J. Kopotic, MSN, RRT, PhDh (Eng).

Critical Care Manager of Clinical and Medical Affairs, Edwards Lifesciences, Irvine, CA, USA.

Takuo’s sojourn into mortality came on February 14, 1936 when he arrived to his parents Monshichi and Tatsu Aoyagi. In 1958, he graduated from Niigata University with a degree in electrical engineering and started work in the medical device industry with the Shimadzu Corporation. In 1971, he joined the Nihon Kohden Corporation, where he completed his life’s work over the next almost 50 years. He received a PhD in engineering from the University of Tokyo with his thesis: “Non-invasive measurement of light absorption in blood based on pulsatile variation of light transmitted through body tissue.”

**Fig. 14:**
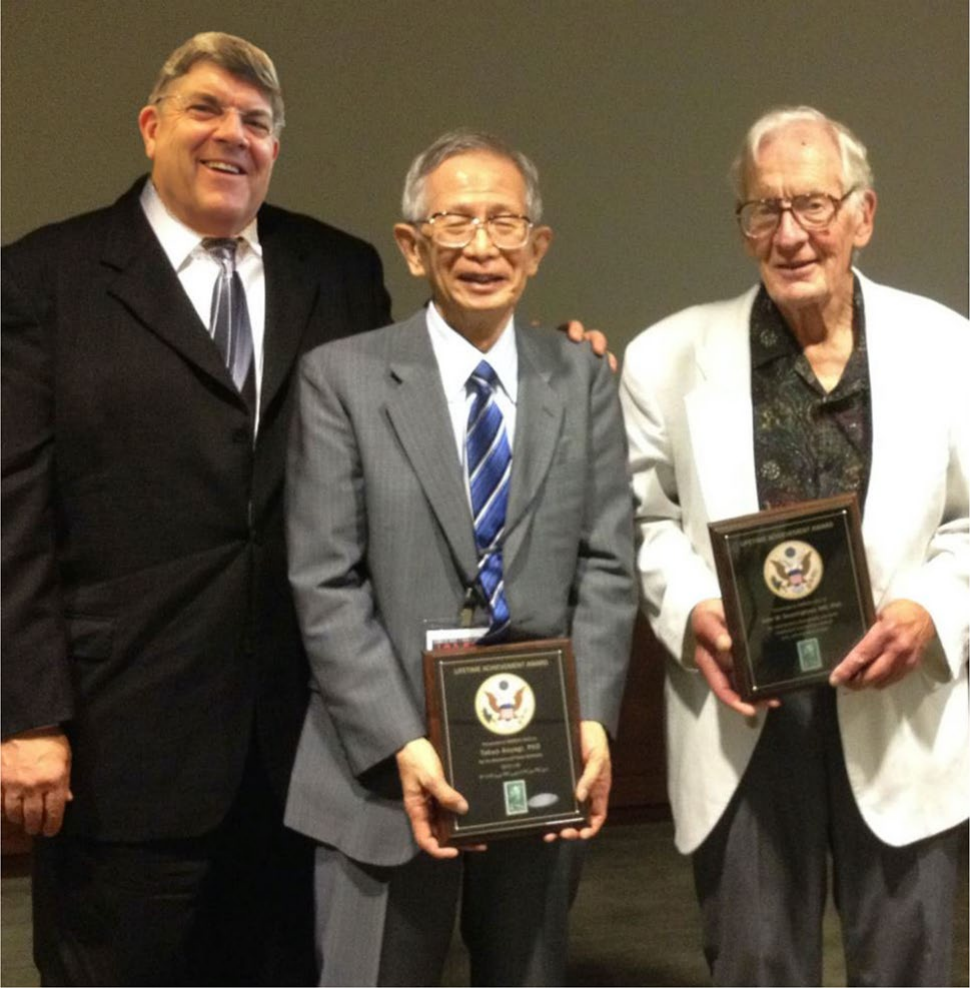
A photo at the Lifetime Achievement recognition during the Innovations and Applications of Monitoring Perfusion, Oxygenation and Ventilation (IAMPOV, 2012) symposium on the campus of Yale University

Dr. Aoyagi has received many accolades and honors but, in my opinion, the finest came from Professor John Severinghaus, who in 1987 met Takuo and then recognized him in the worldwide medical literature for invention of the pulse oximeter. In the attached photo (Fig. 14), Takuo is standing next to me and Dr. Severinghaus is to his side, while they both received Lifetime Achievement recognition during the 2012 Innovations and Applications of Monitoring Perfusion, Oxygenation and Ventilation (IAMPOV) symposium on the campus of Yale University. The following IAMPOV was held at St. Luke’s International University in Tokyo in 2015. The next meeting will be held in London in 2021.

In the proceedings of the 2007 IAMPOV symposium, John Severinghaus walked the attendees through several decades of advancements in optical spectroscopy of the ear, a method that allowed the calculation of blood’s saturation with oxygen but required complex calibration. This work was mainly focused on assessing pilots in flight. Then came Takuo that “was interested in measuring cardiac output noninvasively by the dye dilution method using a commercially available ear oximeter. He found through computation that dividing the absorptions of the red (805) and infrared (930) signals by each other cancelled the pulse noise which prevented measuring the dye washout accurately. He then realized that these pulsatile changes could be used to compute saturation from the ratio of ratios of pulse changes in the red and infrared. His ideas and equations were shared, adapted, and improved by Minolta into the first practical finger type device in 1977, stimulating other firms to further improve and market pulse oximeters worldwide in the mid-1980s.” Severinghaus summarized, “Greatness in science often, as here, comes from the well-prepared mind turning a chance observation into a major discovery.” [1]

I received the news of Dr. Aoyagi's passing from Dr Katsuyuki Miyasaka (“Kats”), who as a physician researcher, worked closely with Takuo for over 30 years. During that same period, I became associated with Kats as we were co-investigators in the NIH-NHLBI, Collaborative Clinical Trial on High Frequency Ventilation in Premature Infants." Contract #NO1-HR-46005 (RFP-NHLBI-84–4; HIFI-Trial). In that study, pulse oximetry data were part of the collected parameters. Kats and I grieved about the loss of a brilliant mind, kind friend, and noble man that had blessed our lives. I then reminisced with Kats about Takuo and his wife (Yoshiko) telling me about meeting someone else of my faith (i.e., the Church of Jesus Christ of Latter-Day Saints). Takuo had a valued relationship with the renowned Dr. Homer Richards Warner from the University of Utah. A cardiologist by training, Homer was the first to develop an electronic medical record system in the US, but he had some experience with the dye dilution method. The two couples stayed at each other's homes on multiple occasions. I remember Takuo speaking highly of Warner's contributions to medicine and Warner reciprocating. Takuo reminded me and my son, that he knew another Christian missionary, i.e., "Elder Homer Warner that came to Japan as a young man." Takuo and Yoshiko mentioned their cherished visits to Homer and Kay Warner in Salt Lake City.

John Severinghaus credited the development of the CO_2_ electrode to Richard Stow (John refined Stow’s design to include a more stable electrolyte, which then became the Stow–Severinghaus electrode used for measuring CO_2_ in blood gas analyzers). Similarly, Takuo gave credit to others for refining pulse oximetry, “I appreciate Minolta. Without their recognition of idea of pulse oximeter, the idea might be buried.”

While his intimate family of survivors include his wife (Yoshiko), and three children (Kaori, Midori and Yasutoshi), so many others have been and continue to be blessed by Takuo’s creation of pulse oximetry and his never-ending curiosity of the interrelationship of engineering concepts related to human physiology, particularly with interactions between heart, lung and vascular function. Sense Takuo’s vision, when with Kats 20 years ago he wrote, “Opening of the first door of pulse photometry gave us pulse oximetry. The next door of pulse photometry opens to multi-wavelength pulse photometry. Dyshemoglobins (e.g., COHb and MetHb) can be measured with a multi-wavelength system. The elimination of artifacts caused by body movement is possible without sacrificing response time. It may also become possible to measure hemoglobin concentration.” [42].

“Dr. Aoyagi hoped to use the oximeter to develop a noninvasive way of measuring cardiac output (the amount of blood pumped by the heart) through a method known as dye dilution, in which dye is injected into a patient. Rather than aid fighter pilots in dogfights, he hoped that his invention would signal a hospital patient’s need for artificial ventilation.” [43]

I wish to take minor liberty with a quote from Sir Isaac Newton, "If *WE* have seen further, it is by standing upon the shoulders of giants." For me that is certainly the case for my apprentice association with Takuo Aoyagi. His engineering efforts raised awareness that ischemia is a precursor of major organ failure and that patients are benefitted through its early detection by monitoring oxygen carrying capacity and perfusion. Dr. Aoyagi was small in height but mighty tall in deed and in humility. I treasure his impact upon my life, many kind words, and thoughtful critiques of ways I could be better personally and professionally.

## Pulse oximetry: The heart of Lifebox’s work—honoring Dr. Takuo Aoyagi

Kitty Jenkin, Head of Communications.

Alex Hannenberg, M.D., Trustee.

Atul Gawande, M.D., M.PH., Co-Founder and Chair.

Lifebox Foundation, Brooklyn, NY, USA.

Dr. Takuo Aoyagi’s invention of the pulse oximeter has saved the lives of millions of people across the globe. After the sadness of his death this year, Lifebox, a non-profit founded by Dr. Atul Gawande with four leading medical organizations to improve surgical outcomes globally, is commemorating his legacy and the evolution that Dr. Aoyagi’s work led to across the healthcare setting.

“It is hard to express the scale of transformation in patient care that resulted from Dr. Takuo Aoyagi’s invention of the pulse oximeter. Effective safety monitoring is an essential part of patient care. Pulse oximeters gave us a way to monitor, with a simple finger probe, the oxygenation of people’s blood. That rapidly made them indispensable and lifesaving across health care - from the operating room and intensive care, to the identification of childhood pneumonia, to the triage of COVID-19 patients. They have become so universal, we can all too easily forget that this transformation in patient care happened in our lifetime. We hope that Dr. Aoyagi’s family and loved-ones will find solace in the millions of lives that have been - and will continue to be - saved throughout the world because of his genius. Our gratitude to Dr. Aoyagi is boundless. On behalf of Lifebox, thank you,”

Dr. Atul Gawande, Lifebox Co-Founder and Chair.

In surgery, the critical innovation of the pulse oximeter was not just that it can display the level of oxygen in the blood, but that it can also do it instantly and continuously, producing a variable pitch auditory signal with each heartbeat. For surgical teams, these features allowed them to “see” whether a patient was getting enough oxygen to survive when under anesthesia. Anesthesia providers often refer to their pulse oximeter as their “eyes and ears”.

In high-income countries, such as Japan and the US, pulse oximeters have been the bedrock of anesthetic care for decades. Unfortunately, due to price and adaptability constraints, pulse oximeters are still lacking from many operating rooms in low- and middle-income countries. In 2011, Lifebox estimated that 77,000 operating rooms around the world still lacked this essential piece of medical equipment. Seeking to address this problem, four of the world’s leading medical organizations joined together to globalize Dr. Aoyagi’s invention. The Association of Anaesthetists of Great Britain and Ireland, Brigham and Women’s Hospital, Harvard T.H. Chan School of Public Health, and the World Federation of Societies of Anaesthesiologists led the way with the creation of Lifebox—a non-profit organization that works to make surgery and anesthesia safer. Lifebox’s founding aim was to close the “oximetry gap” by equipping operating rooms with pulse oximeters.

Today, nearly, a decade later, Lifebox focuses on three core pillars of safer surgery—improving anesthesia safety, reducing surgical infection rates, and strengthening surgical teamwork. By working alongside local partners, it provides the training and tools needed to save lives through safer surgery. All of this work is rooted in the World Health Organization’s Surgical Safety Checklist.

The Checklist is a simple communication tool that has been proven to reduce complications and deaths from unsafe surgery by up to 40%. When the WHO Surgical Safety Checklist was introduced in 2008, the only piece of equipment included on the Checklist was a pulse oximeter—a testament to the international recognition of the undisputed critical role Dr. Aoyagi’s invention has played in improving surgical safety.

When Lifebox launched, it developed a pulse oximeter specifically designed for use in low-resource settings—with robust construction and rechargeable batteries that stay on even when the power fails. To date, more than 26,000 Lifebox pulse oximeters have been distributed throughout 116 countries for vital patient monitoring. The distribution is accompanied by oximetry and anesthesia safety training, recognizing that for many of the recipients, this is their first patient monitoring technology.

“Following a routine cesarean section, I put a pulse oximeter onto the newborn baby as she was wheeled into recovery with the mother. As I prepared for the next patient, I heard the beep of the Lifebox suddenly change pitch; I ran over and saw the alarm going off, as the baby’s saturation started to drop,” explained a nurse anesthesia provider in Sierra Leone who received a Lifebox pulse oximeter. “There was a time in this hospital when this baby would have died because we did not have appropriate oximetry available, but the oximeter is a second set of eyes and ears. Instead, I was able to immediately attend to the infant, and support her breathing. A minute more and it would have been a different story - nine months of labor and an operation ending in tragedy.”

Dr. Aoyagi lived to see his invention become one of the most crucial tools in fighting the COVID-19 pandemic. “Silent hypoxia” is a defining feature of COVID-19, with patients slowly starving of oxygen without the usual shortness of breath that would see them seek care. By the time many COVID-19 patients are having trouble breathing, they are already critically ill. The best tool to detect these patients is a pulse oximeter.

With the start of the COVID-19 pandemic in early 2020, Lifebox pivoted its activities to support healthcare workers to provide safe surgical and COVID-19 care and protect themselves. The mainstay of this response has been the distribution of 6,500 Lifebox pulse oximeters to frontline healthcare providers across 43 countries along with guidance on how to use them in COVID-19 care. Lifebox’s COVID-19 response will last as long as the pandemic continues to devastate health systems and lives, but the core mission—to improve the safety of surgery and anesthesia—remains the same. Pulse oximetry remains at the heart of this work.

There is still much work to be done in the global access to pulse oximetry. Lifebox will expand beyond operating room use to ensure monitoring is available for the full perioperative process—from pre- to post-op—in intensive and neonatal intensive care units, and in the transfer of patients to a higher level of care.

Lifebox celebrates the life of Dr. Aoyagi with every pulse oximeter distributed to a healthcare provider. We cannot think of a more lasting legacy to Dr. Aoyagi than the millions of pulse oximeters in use across the world keeping patients safe.

## Pulse oximeters: The invention that changed the paradigm of patient safety around the world—in summary and in closing this special feature

Katsuyuki Miyasaka, M.D., Ph.D.

Professor Emeritus, St. Luke’s International University, Tokyo, Japan.

### Background of the contributors (bold) and how things developed in Japan: From the inception to the present

Pulse oximeters can be used on all people no matter their color, race, age, body shape, place of measurement, or type of device. By merely turning on a switch, a clear number from 0 to 100% is displayed stably and in healthy people a number that “seems right” shows up. However, according to Dr. Aoyagi, the numbers displayed just “happen to look right,” and to correctly interpret the results, clinicians must also consider the background in which the number gets displayed. In other words, besides the safety, stability and ease of use of the device, it is important not to overlook the precision and reliability of the measurement parameters, and also to understand the physiological and medical issues involved to correctly interpret the number displayed.

Pulse oximeters measure oxygenation, not respiration, but ordinary people and even some medical people tend to overlook this [44]. In patients receiving oxygen during sedation for procedures, a display of 100% is not cause for relief. It is a percutaneous measurement subject to various factors, but is highly reliable when there is no body movement with a good pulse. In cases of extremely low measurements, sometimes, it is better to believe the numbers than the patient’s clinical presentation. It is known by Japanese media that such lack of knowledge can have unhappy results.

As has been seen in the COVID-19 pandemic, there are indications that it is possible for such things to occur, as overlooking silent hypoxia in patients with no symptoms [45, 46]. Dr. Aoyagi was strongly concerned about the lack of understanding of pulse oximeter measurements, even before the devices became popular with the general public. This concern guided his research on establishing a theory of pulse oximetry in his later years. Even if you do not consider the pandemic, it has been reported that it is possible that moderate hypoxia in black people is overlooked almost three times as much as it is in white people [47]. While skin color may not be a problem in Japan, where there is little diversity, it is possible that other such reports will come out from other areas of the world. The best memorial to Dr. Aoyagi, who died before he could finish his work, would be for those of us in clinical research to spread correct understanding of pulse oximetry to medical professionals and society in general.

### Dr. Aoyagi’s contributions and recognition both inside and outside Japan

The inventor of pulse oximetry, Dr. Takuo Aoyagi (1936–2020), died on April 18, 2020. He was 84 years. The first case of the new corona virus (COVID-19) in Japan was found in mid-Jan., 2020 and seemed to show signs of being rampant in mid-February. In hindsight, the peak of the first wave (500 cases nationwide) just happened to coincide with Dr. Aoyagi’s demise. Vaccinations in some countries finally started at the end of 2020, but at that point about 83 million people around the world had been infected and 1,850,000 people had died [48]. It is thought that pulse oximeters were used in almost all these patients, many outside medical facilities. Dr. Aoyagi did not live to see that day. It is regrettable that he left us before he could complete his work on multi-wave theory and its precision and reliability.

**Fig. 15:**
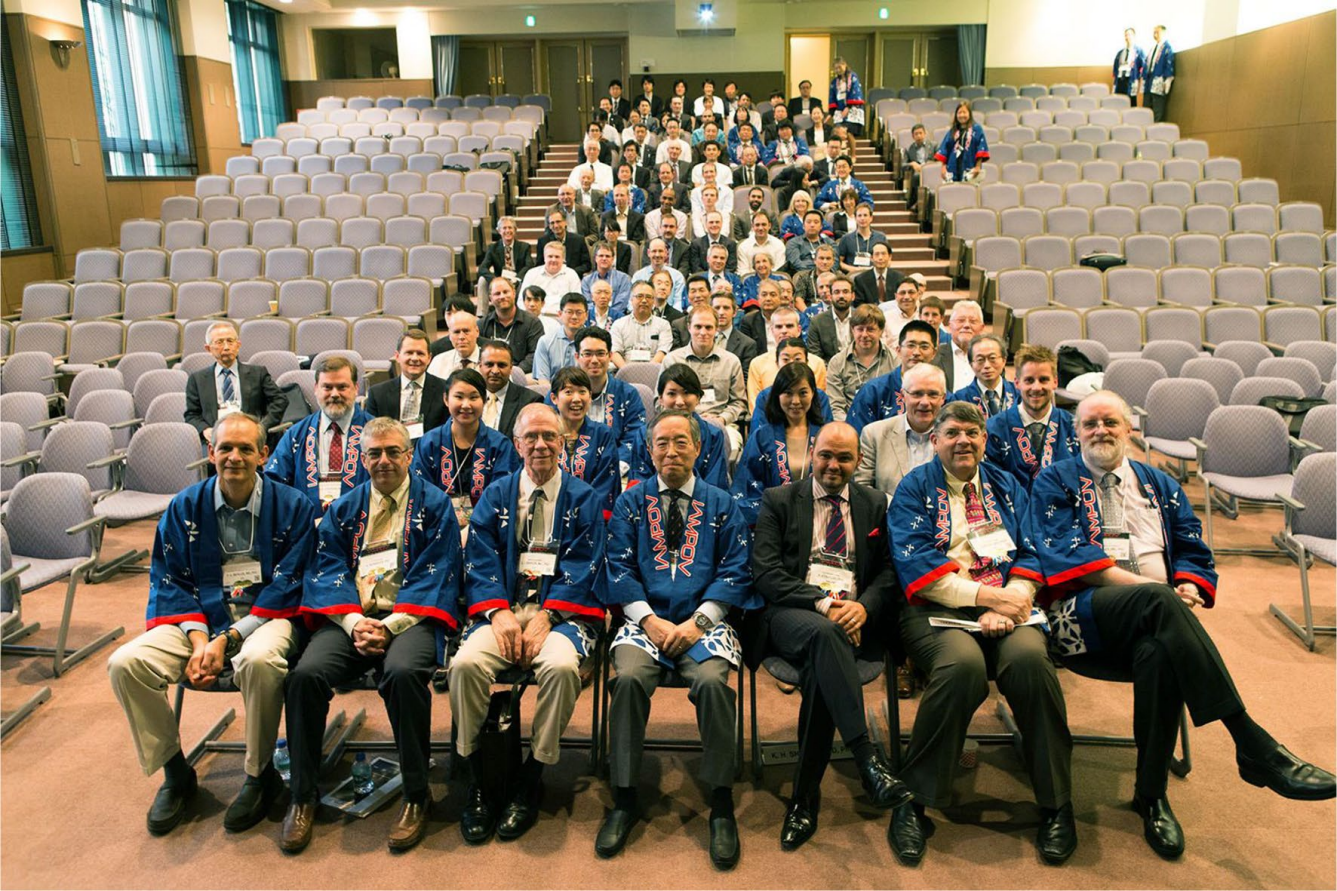
Memorial photo in 2015 Tokyo IAMPOV Symposium (at auditorium in St. Luke’s International University)

Dr. Aoyagi’s passing was reported immediately and widely by foreign media, such as the New York Times (US) [49], Washington Post (US) [43], Globe and Mail (Canada) [50], and CNN (worldwide) [50]. I was struck by the sharp contrast between the high interest shown by those overseas with reporting in Japan [51, 52]. I have attended quite a few conferences overseas with Dr. Aoyagi and have been moved by how researchers around the world would come up to him each and every time to thank him for his invention. I also feel how inadequate was the recognition he was given in Japan. Against this background, the 4^th^ IAMPOV (Innovations and Applications of Monitoring Perfusion, Oxygenation and Ventilation) international symposium was held in Dr. Aoyagi’s home ground, Japan. With the support of the Japan Association for Clinical Monitoring and other groups, a group of approximately 180 enthusiastic people (60 from abroad) gathered in Tokyo. It was a moving experience for 3 days, to see a group of people from all over the world, interested in the same issues, gather and discuss our research freely, with no restraint, regardless of specialty or affiliation (Fig. 15).

Contributors from IAMPOV to this memorial issue are **Dr. Bob Kopotic**, from the secretariat, and **Dr. Kirk Shelley**, professor at Yale and the organizer of the 3^rd^ IAMPOV in 2012. **Dr. Kirk Shelley**, told us how he nominated Dr. Aoyagi for the 2013 Nobel Prize in Physiology or Medicine. Unfortunately, with his death, Dr. Aoyagi will not get the Nobel Prize, but I would like to express my gratitude and respect for Dr. Shelley’s courage in telling us what happened, so that it will not disappear from history. Dr. Aoyagi has received many honors. In 2015, he was awarded the prestigious IEEE (Institute of Electrical and Electronics Engineers) Medal for Innovations in Healthcare Technology (as prestigious as the Nobel Prize in his field). The American Society of Anesthesiology (ASA) voted to give him honorary membership, one of their rare honors, in 2020. ASA will present the award in his memory at their annual meeting in October 2021. On Dec 25, 2020, he was posthumously awarded the 4^th^ Grand Prize for Medical Research and Development by the Prime Minister of Japan. **Mr. Hirokazu Ogino**, the CEO of Nihon Kohden received the award on behalf of Dr. Aoyagi. This is the most prestigious award in Japan in this field [53]. It can now be said that the reputation of Dr. Aoyagi in Japan has finally caught up to what it is overseas.

### Contributions of pulse oximetry to patient safety

In addition to the great recognition Dr. Aoyagi had previously (IEEE Award and nomination for the Nobel Prize), many others from abroad joined in to add their voices to his memory: **Ms. Kitty Jenkin** of LifeBox, and five other individual contributors. One of the founders of the LifeBox Foundation was Dr. Atul Gawande, who was a pioneer in the development of the now indispensable Surgical Check List. The foundation works to make pulse oximeters available to operating rooms around the world. **Ms. Kitty Jenkin**, in charge of communications at the foundation, has contributed to this memorial issue signed by Dr. Gawande and all the members of the board, representing a wide spectrum of countries and backgrounds.

Eight Japanese people who have worked with Dr. Aoyagi directly have added their articles, so that we cover Dr. Aoyagi’s career from his initial ideas to what we know of him today. I believe that we have covered all the steps of the development of pulse oximeters and the important roles other people played. Fortunately, most people have clear memories and impressions, and I’m sure Dr. Aoyagi would have been very proud of what they wrote.

### Pulse oximetry: Two beginnings

The invention of pulse oximetry started in Japan and is now used in both medicine and by ordinary people around the world. Surprisingly enough, two patents were filed at almost the same time in 1974. Dr. Aoyagi, on behalf of Nihon Kohden (patent filed March 29, 1974) and **Mr. Akio Yamanishi**, on behalf of Minolta (patent filed April 24, 1974) came upon this idea completely independently. Dr. Aoyagi’s device, that came first, used a dye densitometer on the earlobe to measure cardiac output. He came upon his idea during an experiment to eliminate superimposed pulsation noise. His light source was an incandescent lightbulb, and his point of measurement was the earlobe, both of which made it difficult to develop a practical device and the project ended. Chances are that it was not pursued because the invention was a side product and did not align with the company’s main project.

Dr. Aoyagi reported on his invention to his supervisor, and it just happened that a physician the supervisor was visiting heard about it and work on a prototype was started. They were less interested in the significance of oxygen saturation and were mainly looking at new methods of measurement. Dr. Aoyagi told me that once the paper was published, there was no more mention of turning it into a clinical device. I do not know what really happened, but the result is as we know it. However, Aoyagi continued his research into establishing a theory of measurement through the years, and after a break of about 10 years, Nihon Kohden renewed its development. They allowed Dr. Aoyagi to pursue his research until the end and Dr. Aoyagi fulfilled their expectations.

On another front, Mr. Akio Yamanishi’s group was taking advantage of the new LED technology to develop fingertip plethysmography, etc. and the development of a pulse oximeter was one of their primary projects. They succeeded in developing the world’s first fingertip pulse oximeter. Two contributors to this issue, **Dr. Ikuto Yoshiya** (Anesthesia Professor at Osaka University at the time) and Dr. Yasuhiro Shimada (Assistant Professor at the same University) were involved, but their contributions were limited to improving precision through analysis. Minolta started selling their device (OXIMET 1471) through Mochida Pharmaceuticals in June 1977, but rather than using LED as a light source, they used a combination of tungsten and fiberoptic cable, so although the device was usable, it was difficult to operate. It is possible that the red spectrum in LEDs at the time was not sufficient, but it was an unfortunate choice.

I would like to note that there was absolutely no sense of competition between Nihon Kohden and Minolta at the time. Minolta had been pursuing this project since the late 1960s at the time of the Apollo Project, so as part of the company’s protocols, they began to apply for a patent for Yamanishi’s group at the end of 1973. Dr. Aoyagi’s application, sent in before his presentation at a conference, just happened to arrive at the patent office a few weeks earlier. I can only imagine how shocked the Minolta group must have been. Dr. Aoyagi’s patent was restricted to within Japan, but Minolta obtained an international patent. I do not have detailed patent information and I have no idea how latecomers managed to overcome patent issues, but it was good for mankind that Dr. Aoyagi’s patent did not restrict the sound competition of pulse oximeter development.

**Fig. 16:**
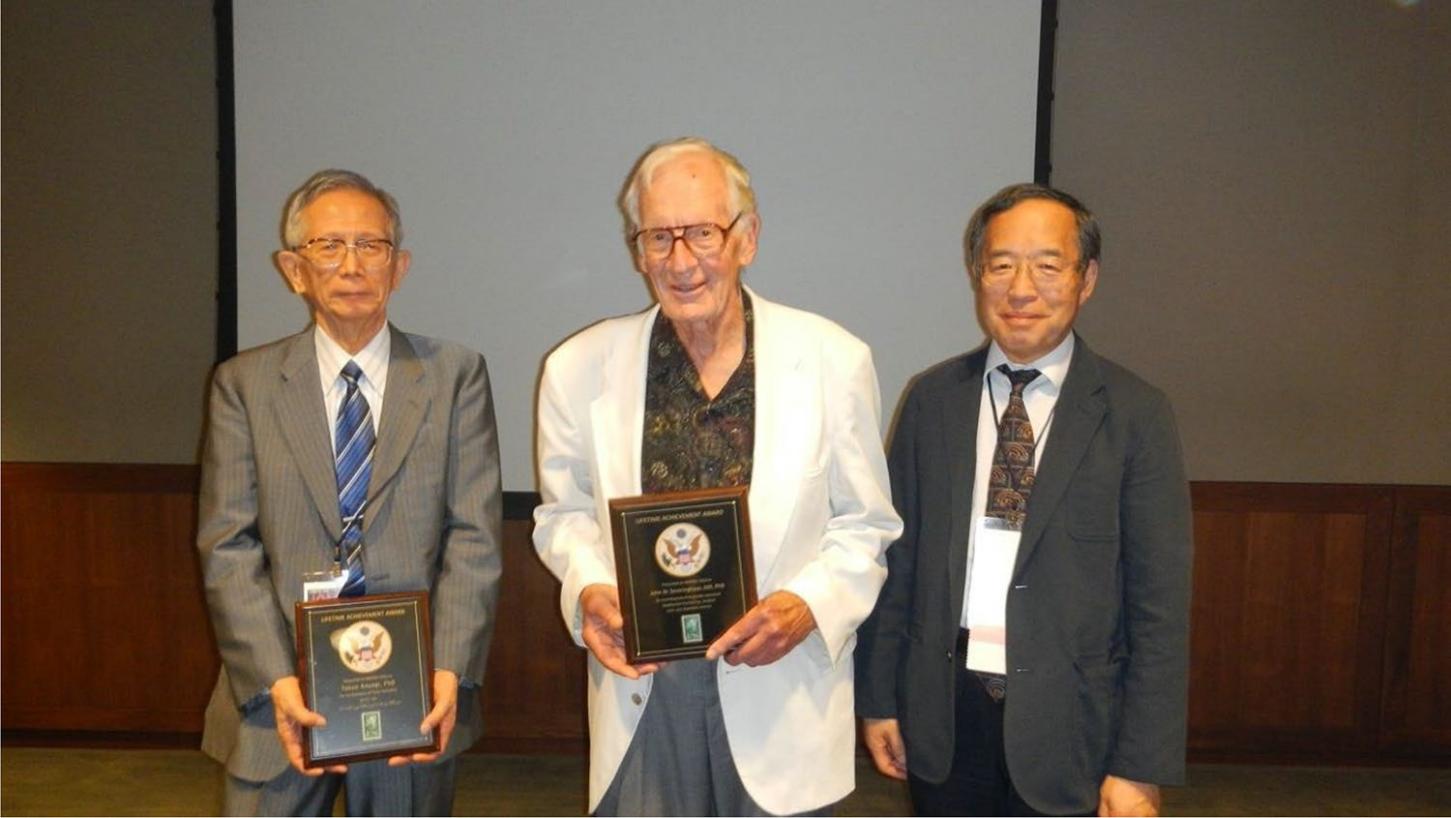
Memorial photo at the 3rd IAMPOV Symposium Award Ceremony, 2012

Most of the information on Dr. Aoyagi is only in Japanese, and his company, Nihon Kohden, did not try to develop the product. In 1987, Dr. John Severinghaus (Fig. 16) discovered Aoyagi’s achievements and made him known to the world, including Japan which was unaware of his invention. The first serious published paper on pulse oximeters in English was written by **Dr. Yoshiya** in 1980 [11] and influenced researchers and inventors all over the world (Pierce EC: ASA The 34^th^ Rovenstine Lecture, 1995). This history is already well known, and on behalf of Dr. Severinghaus, who is advanced in years, **Dr. Robert Kopotic**, a central figure in IAMPOV, has written some of this history for us.

### Clinical significance unrecognized in Japan

The OXIMET 1471 pulse oximeter that went on the market in 1977 seems to have been reviewed by several university academic anesthesiologists in Japan. However, while the device was judged to be useful at some level as a research measurement device, it seems that either there were no suggestions to help it spread as a clinical device or that if there were, they were not heeded. Only 200 devices in total were sold. Dr. Kunio Suwa (Associate Professor of Anesthesiology at Tokyo University) tried it out in 1992 on his own volition, but unfortunately even then, as now, a structural problem existed in Japan’s medical device industry.

The very first time Dr. Aoyagi presented his invention, the pulse oximeter, to Japanese anesthesiologists was in 1989 at The Japan Society for Clinical Anesthesia Academic Meeting in Tokyo. At that time, most anesthesiologists in Japan had heard of Nihon Kohden, where Dr. Aoyagi worked, but it was not until 2002 when the Japanese Society of Anesthesiologists gave Dr. Aoyagi an award for his contribution to society, that his name and Nihon Kohden’s pulse oximeter became familiar to anesthesiologists in Japan. The first person to tell the world about Aoyagi’s achievements was, as mentioned above, Dr. Severinghaus of UCSF [1, 5]. Dr. Severinghaus himself has made great contributions to the field of blood gas analysis and is known for his development of an electrode for partial pressure measurement of CO_2_.

### Minolta’s OXIMET-1471 pulse oximeter goes to the US

A large Japanese trading company tried to sell Minolta’s OXIMET-1471 pulse oximeter in the US, and in connection with this, they offered use of the machine for evaluation to an anesthesiologist (Dr. Charles Whitcher) at Stanford University in 1977 and it was used clinically [12]. The development team at Minolta focused on scientific analysis of the tendency to overestimate at low oxygenation levels, but they were not particularly interested in making improvements to make the machine easier to use on patients in a clinical setting.

**Fig. 17:**
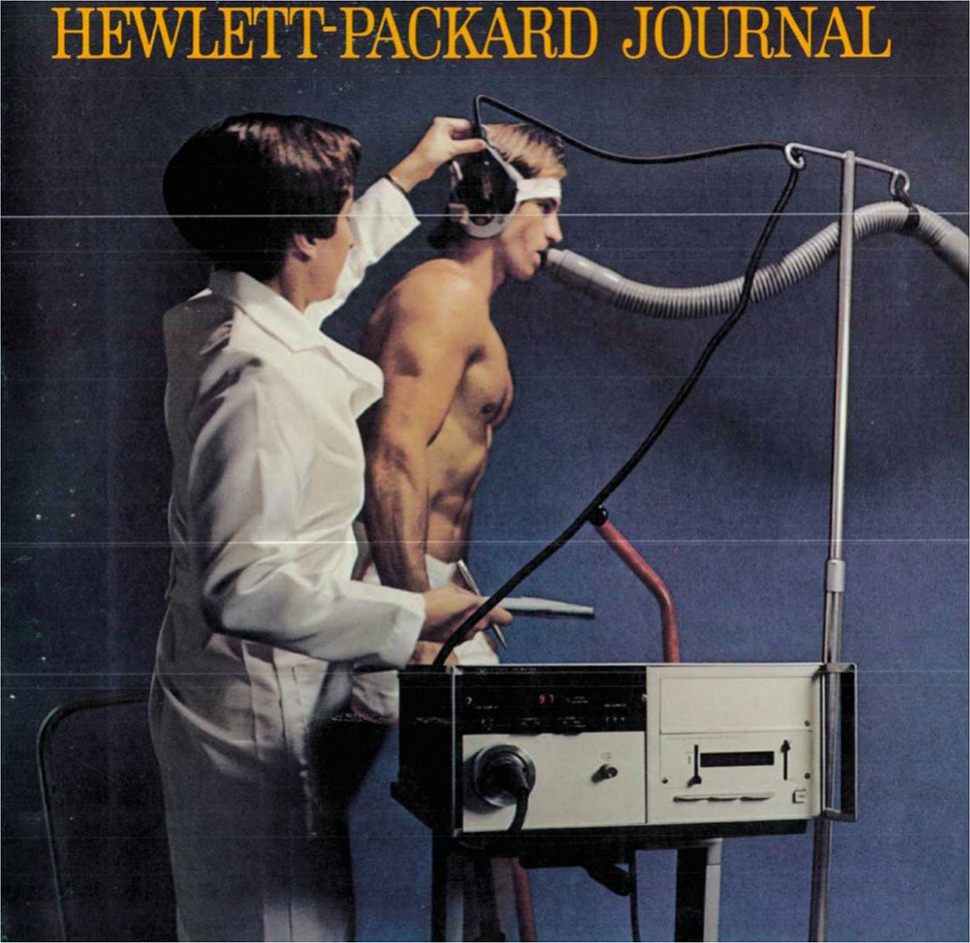
HP Company ad for an ear oximeter (1973). Eight wavelengths (650-1,050 nm), heated ear probe to obtain arterialization. It was accurate, but large and hard to operate. It was not a monitor.

However, this offering turned into an opportunity as it can be thought that Dr. William New saw the device. Dr. New, who later founded Nellcor and who used to work for HP as an engineer, played an extremely important role in the development of the currently used clinical pulse oximeter. Dr. New is an anesthesiologist who has a background in electrical engineering and biology. He was working in the same hospital as Dr. Whitcher at the time. Dr. New was very familiar with the HP ear oximeter (Fig. 17).

In 1980, Professor Whitcher’s group reported on the Minolta Oximeter Model 101 (OXIMET-1471) at the Annual Meeting of the American Society of Anesthesiologists (ASA). They clinically evaluated the device on 5 healthy male subjects and indicated the possibility of its being a clinically effective way to pinpoint the tendency to overestimate at low oxygenation levels. Their abstract quotes from an article by Suzukawa in 1978 (translated into English), but the presentation at ASA was not published.

Corning Medical was also involved in this research as they had just started to sell an invasive blood gas analyzer in Japan. I had a chance to visit Corning in October 1982 for a different reason and I was asked about it directly, but it is unclear how the relationship between Corning and Minolta developed afterward. This story is from the year before Nellcor started selling the N-100.

Minolta contributed to the initial spread of pulse oximeters and OXIMET-1471 was good enough. Minolta played an important role in the initial spread of pulse oximeters and OXIMET-1471 was equipped with very high standard technology. The precision was at research level and impressive, but without the system needed for Japanese anesthesiologists to give appropriate feedback on its usefulness as a clinical device, there was no way to improve it enough to take advantage of being the front runner.

### Nellcor appears on the scene

Nellcor was founded in 1981. A close copy of the fingertip device by Minolta was made into the prototype N-100A in 1982 and was being sold in 1983 [54]. It was significantly and overwhelmingly an excellent device in terms of performance, design, operability and clinical sensibility. A rise in the call for safety awareness in anesthesia also helped the device spread rapidly. The founder of the company himself was an anesthesiologist, but advice from pediatric anesthesiologists was taken into account during development. The latest and most important pulse oximeter for anesthesia to hit the market, and the one that played a major role, was Nellcor’s. Dr. William New, its founder, passed away in 2017 and unfortunately, none of the people close to him that I contacted were available to write an article for this memorial.

With great luck, I learned that **Dr. David J. Steward** (at that time at the Hospital for Sick Children in Toronto) had been asked directly by Dr. New to evaluate the prototype Nellcor N-100A. He agreed to write about what he knew and submitted an invaluable photo of the prototype N-100A. I knew Dr. R. Raphaely (staff at the pediatric anesthesia department of the Children’s Hospital of Philadelphia), the person who introduced Dr. New to Dr. Steward. I knew most of what happened after, but this was the missing link! The secret to the success of the Nellcor N-100 lay in the input from top level clinical pediatric anesthesiologists. There is no doubt that the spread of pulse oximeter use in a clinical setting was due to the proficient efforts of Nellcor in making early and skillful use of recently matured LED technology from Japan and putting everything together to make a practical clinical monitor.

There is also another factor, the raised social awareness of the number of malpractice cases charged against anesthesiologists in the US and the high cost of malpractice insurance for anesthesiologists in the US in the 1980s. Above all, in 1986 the ASA Guidelines for anesthesia monitoring included the use of pulse oximeters as one possible choice. These Guidelines spread, not just in the US, but all over the world and were a big turning point for anesthesia safety.

### Increased momentum for awareness of anesthesia safety in the US

There were many factors in the movement to improve patient safety during anesthesia, boosted by Cooper’s study in the late 1970s [55]. The high cost of malpractice insurance for anesthesiologists led to the discussion of formulating the “Harvard Guidelines,” drawn up by a group of people from the hospitals associated with Harvard University (1983). Other factors were ABC’s television documentary, “The Deep Sleep: 6,000 Will Die or Suffer Brain Damage”(1982), the establishment of APSF, and incorporation of the ASA Guidelines. **Dr. Jeffrey Cooper**, known as the Father of Patient Safety, was involved in the formulation of the “Harvard Guidelines” and has written an article for this memorial.

He discusses the role played by numerous factors, such as the possibilities of non-invasive measurement (inventions), the development of excellent devices based on human engineering (Nellcor N-100), and education about the clinical significance of monitoring (ASA Monitor Standards).

Japanese companies were left behind as they were unable to fathom the wave of the times. They could not see the importance of the background of devices, in other words, it was not just technology that was important, but the role of the people for whom the device is being created. There was a lack of understanding of the importance of such factors as cooperation between the developers of medical devices and the clinical setting, including social aspects. There was also a lack of understanding of the entire production process, including education and how products reach clinicians and are applied (concept of BioDesign).

When the Harvard Guidelines started to be discussed in the early 1980s, one of the most important missions was to establish rules that anesthesiologists would have to adhere to, even in the country of the free. Discussion was focused on fundamentally important issues that no conscientious anesthesiologist could argue, such as having anesthesiologists be physically present, rather than introducing still arguable monitoring devices.

Thus, although incorporating the use of capnometers or pulse oximeters in the guidelines was discussed, they were eventually left out, because they were still in the early stages of development [56]. The ASA Anesthesia Standards from 1986 recommend the use of oxygenation monitors, but the requirement to use pulse oximeters did not come until 1989 when the standards were amended. Up until this point, the issue of anesthesia safety was centered mainly on activity in the US, just as Nellcor’s N-100 was playing a central role in the market.

However, in 1984, the British giant BOC/Ohmeda procured an American firm Biox and new activity began in England. Biox products were widely used in Japan and played a central role.

### Influence of Biox/Ohmeda

The American market for oximeters started in 1979 with Biox’s (Denver) device, Biox II. According to Dr. Jonas Pologe (personal communication) who was involved in development at Biox, the device, an ear oximeter, was made after considerable study of both Aoyagi’s and Minolta’s published reports. I did clinical research on Biox III in the latter half of 1984 in Japan and found it to work in a clinical setting with good performance. I reported on this research in 1986 [57]. It was a stable and convenient patient monitor during anesthesia, and while it was better than Minolta’s or Nellcor’s devices, it was primarily a research device designed for respiratory internists. With little input from clinical anesthesiologists at Biox, Nellcor’s device, that came after, was clearly and overwhelmingly better for clinical use.

In 1984, Biox was bought by the British Company BOC, a world leader at the time in anesthesia related products. The fingertip model, Biox 3700, was introduced [41]. In anticipation of selling in Japan, they sought input from Japanese sales offices and anesthesiologists. Improvements, such as waveform display and the shape of re-usable probes, were made. The efforts by **Mr. Yasuhiko Sata** (Tokibo) to use sales techniques unique to Japan were successful, and in Japan, Biox sold much more than Nellcor that had already captured the anesthesia market in North America and Europe. At this point, neither Nihon Kohden nor Minolta had products that were better than foreign ones. The domination of the Japanese market by foreign brands continued.

### Widening interest in research

Approximately 50 world leaders in physiology and anesthesiology, including Dr. Jim Payne and Dr. John Nunn from England and Dr. John Severinghaus and Dr. Kevin Tremper from the US, gathered in Chartridge, a suburb outside London in May 1985. From Japan, Dr. Kunio Suwa (Tokyo University, Anesthesia Department) talked about the oxygen dissociation curve [58], and Dr. Katsuyuki Miyasaka (National Children’s Hospital, Anesthesia Department), the sole pediatric anesthesiologist there, talked about his clinical experience with tragus probes adapted from adult earlobe probes for Biox III in pediatric patients [57]. The conference provided an opportunity to discuss a wide range of issues from the definition of SpO_2_ and the categorization of oxygen saturation, to the clinical usefulness of pulse oximetry and the significance of biological research.

In response to this, in 1986, Dr. Kunio Suwa (Tokyo University, Associate Professor), **Shosuke Takahashi** (Kyushu University, Professor of Anesthesia) and Dr. Hiroshi Sankawa (National Children’s Hospital, head of the Anesthesia Department) were the primary movers to start the Japan Research Group on Pulse Oximetry. In 1987, **Dr. Katsuyuki Miyasaka** (National Children’s Hospital, Department of Anesthesia) organized an international conference (Hakone Conference) to discuss the use of pulse oximeters primarily in neonatal medicine. Sales of the Biox 3700 in Japan suddenly started to grow in the fields of anesthesia and NICU. **Dr. Hiroshi Nishida** (Tokyo Women’s Medical College, Professor Emeritus) recalls the important role that pulse oximetry played in increasing a healthy interest in such hot topics as prevention of retinopathy of prematurity, lung surfactant replacement therapy, and high frequency oscillation (HFO).

### From anesthesia to critical care

The Japanese Society of Anesthesiologists created their first safety guidelines (Monitoring Guidelines for Anesthesia Safety) and recommended use of pulse oximeters during anesthesia. This was 7 years after ASA released their first Monitoring Guidelines for Anesthesia in 1986 in the US. **Professor Shosuke Takahashi** played an important role, but more than half of physicians who engaged in anesthesia did not have access to even one pulse oximeter in their institutions. Domestic competition was practically non-existent. Interest in pulse oximeters grew rapidly in the field of anesthesia, but when their use expanded from during anesthesia when patients did not move to the recovery room, ICU and general wards, a big problem arose in how to deal with false alarms from body movement. When venous waves are superimposed on pulse waves, the convenient assumption of pulse oximeters that all pulsation is arterial pulsation no longer holds. In efforts to decrease false alarms, many strategies were tried, such as temporarily freezing alarm information, prolonging the moving average time of the data, and extraction of the arterial waveform during synchronization with electrocardiograms, but none of these served as a fundamental solution.

A group from the Masimo Corporation, founded in 1989 by **Mr. Joe Kiani**, focused on the problem of false alarms from body movement (in other words how to deal with body movement and low perfusion) in pediatric patients with heart disease who tend to be agitated and move a lot, and unavoidable body activity in the PICU (Pediatric Intensive Care), as compared to adult ICUs or NICUs.

### Emergence of Masimo Corporation

From the early 1990s, the Masimo Corporation, looking into cooperation with the major electronic company NEC, found little interest in the problem of body movement in the field of anesthesia in adults, so they developed deeper relationships with **Dr. Katsuyuki Miyasaka** and Dr. Yasuyuki Suzuki from the Intensive Care Department of the National Children’s Hospital (now the National Center for Child Health and Development, Tokyo). They were studying the reliability of and problem of false alarms in respiratory monitors in pediatric ICUs and respiratory therapy in home care pediatric patients. They had also introduced a project called the “Sound of Silence” to address the problem of alarm fatigue in pediatric anesthesia and pediatric ICUs such that all alarms were silenced within 3 times of beeping. Thus, they were able to obtain many hours of raw data and video recordings from pulse oximeters and patients. It was not a comparative study, and it was not published, but this data on Japanese patients in pediatric ICUs helped strengthen strategies to deal with body movement, and thus low perfusion in adults. It helped in improving of Masimo SET (ver. 2.2) that functions well even in patients who move (2000) [28, 59].

Even without a theory of pulse oximetry, they developed a method of measuring SpO_2_ using arithmetic chips and powerful statistical methods. They further added multiple wavelengths and are introducing new monitor indices, such as carboxyhemoglobin and other abnormal hemoglobins, total hemoglobin, oxygen reserve index (ORi), and various circulating blood volume indices. They are pioneering a road of their own. This is a little different from big data analysis, but by proceeding from special extractions and relative relationships, they found causal relationships and I hope this will result in the establishment of a theory.

### Nihon Kohden starts development of pulse oximeters again; problems with multi-wavelengths and precision

In 1984, Dr. Aoyagi was put back in charge of the development of pulse oximeters at Nihon Kohen. Dr. Aoyagi formed a team including Mr. Masayoshi Fuse and Dr. Naoki Kobayashi. As opposed to the clinical, practical approach that helped Masimo succeed, Nihon Kohden followed a conservative, theoretical approach to achieve great precision.

The theory of multi-wavelengths (5 wavelengths) was proposed in 2008 [60] and was established by Dr. Aoyagi in 2015, but they are still verifying the theory and no product has been made. However, this cautious approach is not necessarily a failure to act. A long-term issue for the prevention and survival prognosis of neonatal retinopathy was about the need to adjust the threshold for low perfusion or adjust the fine calibration curve [61]. In 2020, the topic of the clinical significance of measurement differences due to racial differences (skin color) [47] came up, but there was little basis for discussion, because there was no theory and no way to compare numbers using a standardized calibration. The only conclusion is that we’ve reached a limit. In other words, conveniently neglecting differences in skin color, race, adults, infants, body shape, place of measurement, device, etc. is not questioned for the sake of convenience. It is impossible to standardize calibration using actual measurements on human beings who cannot be standardized (no more than calibration can be standardized), between manufacturers and devices, different probes, etc. The road laid down for us by Dr. Aoyagi is of great importance to break the deadlock of the acceptance of differences of 1–2% especially in the low SpO_2_ range and to establish a theory, as emphasized by Nihon Kohden.

### The challenge of in vitro calibration

Dr. Aoyagi and I have known each other since 1980. In our work for ISO TC-121 SC3 (mainly patient monitor devices), we came to be concerned about the precision of pulse oximeters that were being produced without regard for the lack of a method for standard calibration. As ISO searched for a solution for a standard, I suggested incorporating the device into an international standard, without fully understanding the reason for why there was no calibration standard. I ended up being put in charge. Fortunately, Mr. Yamanishi and Dr. Aoyagi were able to participate in the project. At the time, I was using a small roller pump for development of the world’s smallest ECMO for pediatrics [62], and I figured out that if I clipped a probe onto the circuit, I could get decent pulsation. Without sacrificing any people, I thought I could calibrate under extreme hypoxic conditions.

“We might have a principle of pulse oximetry, but no theory.”

When I told Dr. Aoyagi my idea, he said in no uncertain terms “That’s pointless, pulse oximetry has a principle, but it does not have a theory.” Struck by this Zen-like opinion, I was deeply shocked. In the 40 years, since I have known Dr. Aoyagi, he never used strong words like that again. After a short discussion, I said, “Well, it’s your job to establish a theory or whatever. If you do not, you will not get a Nobel Prize.” In response to my young arrogance, his manner fell apart and he said, “Aah. You won that one.” Then he gave a carefree smile, and with a gesture of friendship, stopped the argument, and continued his considerate explanation with a pencil.

### Research on multi-wavelengths

In the end, I never succeeded in establishing an in vitro calibration method for ISO [63], but this was the same as saying the theory had not been established. The most recent ISO standard ended up mandating empirical calibration using blood sampling in healthy adults who are exposed to a non-physiological level of hypoxia. Thus, the accuracy of currently available pulse oximeters ignores such factors as race, age (adult or child), or individual devices. **Mr. Hironami Kubota** questions whether regular household devices really need to go through such a complicated calibration process. It is a very complicated issue.

Dr. Aoyagi started working on a complete theory and after verification with experiments with multi-wavelength simulation models that took into account light scattering and pulsation, and also the effect of surrounding tissue, he presented his work at IAMPOV 2015 (Tokyo) [13]. The main reason for his studies using multi-wavelengths was to improve precision. However, since he was not looking at such factors as abnormal hemoglobin, I think it is possible his research was not considered important enough to result in product development and never became a major project.

### Research that was a stretch

Against this background, Dr. Aoyagi continued his experiments, where a small number of subjects held their breath to create a state of hypoxia, sometimes for a long time. He came to my research lab at the hospital when he felt there was a chance that it would help to have a doctor around. The subjects usually were himself and Mr. Masayoshi Fuse, a long-time member of his team. Over 70 years of age, he felt the overstretched research was ghastly. As a result, the issues for Dr. Aoyagi’s multi-wavelength theory have not yet been fully verified or followed up. As someone who represents the researchers continuing Dr. Aoyagi’s work, **Mr. Kazumasa Ito**, who worked on Dr. Aoyagi’s later research, is committed to solving this difficult problem, and has submitted an article to this memorial issue that shows his dedication to this cause.

### The spread of pulse oximeters in society and the issues involved

While Dr. Aoyagi was pleased with the widespread use of pulse oximeters, he feared that without a theory, the number displayed might take on a life of its own. It is not possible to rule out the influence this strong reluctance Dr. Aoyagi had about believing the reliability of the number displayed had on how he did not come up with a product. But under the shadow of the great usefulness of the device for COVID-19, it is a concern that pulse oximeters are being used not just in the operating room, but everywhere, by medical professionals and ordinary people alike, without a proper understanding of what it means.

**Mr. Hironami Kubota**, who once worked at Nihon Kohden at the same time as Dr. Aoyagi and engaged in the development of central patient monitoring systems, has added a discussion to his article in this memorial issue about how the use of pulse oximeters, developed for use on seriously ill patients in a special environment, does not match its current use elsewhere, from general patient wards to the non-medical general population. While pulse oximeters are classified as medical devices requiring regular maintenance in Japan, they are being used widely by the general population without being aware of what they are, or even escaping safety rules by being embedded in a large number of products. Their performance is improving as a common device and the difference between pulse oximeters for medical use is hard to understand. Noninvasive patient monitors, such as pulse oximeters in wearable form, will continue to flood the market and influence our daily lives. They cause little harm as electronic devices, but if you misinterpret the numbers displayed, critical harm can result. The current regulatory system to protect users from suffering this kind of harm is inadequate.

We, as clinicians, have to inform people of possible dangers from these devices. It is becoming more and more important to let people know how to interpret the number displayed. Although the device is not recommended for running or for measurements other than on the fingertip, they are used without a second thought if the number displayed looks right. There is no problem legally speaking if the number displayed is not on a device labeled for medical use. In the current state of affairs, where the appropriate use of pulse oximeters is not guaranteed, people will not even be able to tell if a device is poorly made as long as the number looks right. When something that can be used by anybody by just clipping on a probe and reading a number, combined with the situation, where pulsations of healthy people are strong, any product will show “normal” values. Even if there was a dangerous condition, no one would notice a problem as long as the number is within the “normal” range.

Even if you do not call it a diagnosis, interpreting the number shown, or particularly judging the line between normal and not normal, requires medical understanding. While it is necessary to educate users about correctly understanding the number, the regulations demanding proper education of the general public in Japan are vague. The manuals included in the devices say “seek a doctor’s opinion if there is a problem,” but this warning is of no use to the lay public, because as things are now, there is no way for them to know if there is a problem or not. However, people use the device to “find a problem.” Thus, the user is left believing in the device without adequate understanding and no one including the company or government has responsibility for misuse of the device.

While the authorities in charge may be interested in the safety of electronic products, they are not interested in how the numbers displayed are interpreted or in the safety of the medical device embedded in the device. There are very few cases, where clinicians are involved in product inspection. Our mission is to educate the public whenever we have a chance and provide them with the knowledge they need to evaluate products, where medical quality and non-medical quality products are combined.

### Epilogue

After participating in this memorial issue and reading all the manuscripts, I feel great gratitude for the immense contribution Dr. Aoyagi made to human health. When I think how the lives of so many people have been saved and how many more will be saved in the future, I feel incredibly fortunate to have lived and worked with Dr. Aoyagi. In 2000, I co-authored a paper, “Theory and applications of pulse spectrophotometry,” which raised my expectations as a clinical researcher for the future of pulse oximetry in the next generation of patient monitors. I realized how privileged I was to have helped develop pulse oximetry for 30 years, but I felt how much regret Dr. Aoyagi had for leaving his task unfinished, while the rest of us continued to treat cavalierly the lack of the true basis of measurement, the theory. Planning for this memorial issue has helped me realize that on the other side of his great contribution to clinical medicine, was the limited number of clinicians directly involved.

It is not that Dr. Aoyagi was untalkative or anti-social, but he definitely did not talk much about things other than his research. I knew him for the better part of 35 years, but even if we talked a bit about art, I do not remember ever talking to him about his family or him personally. I guess I too belong to an era of Japan where that was not unusual.

I must make special note of the fact that I never once stepped into Dr. Aoyagi’s research lab. He always came alone to the room where I worked in the hospital, waiting to talk to me in between patients. Sometimes, he would let me know ahead of time and send documents for me to read, and sometimes I had the chance to hear him discuss things for hours. That time was so special, but I could not share it with my staff, because it was not possible to fit it in around our work schedules. Truthfully, I did not know what Dr. Aoyagi’s position was in his company or what his research and development goals were. I do not think he had the opportunity to let his supervisors in his company know what was really on his mind. The fact that it was not possible to create a structure to join the forces of a top-class research developer from a privately-owned company with a physician like me, working in a national hospital, is typical of the limitations faced by scientists trying to develop new medical devices in Japan.

There is a distinct difference between evaluating “finished” pharmaceuticals and “unfinished” developing medical devices. How to apply medical devices to patients is of paramount importance in medical device development. It is essential for the user and developer to work together in developing new medical devices. Unfortunately, even now there is no such system of cooperation in public hospitals in Japan, rather it is restricted.

**Fig. 18:**
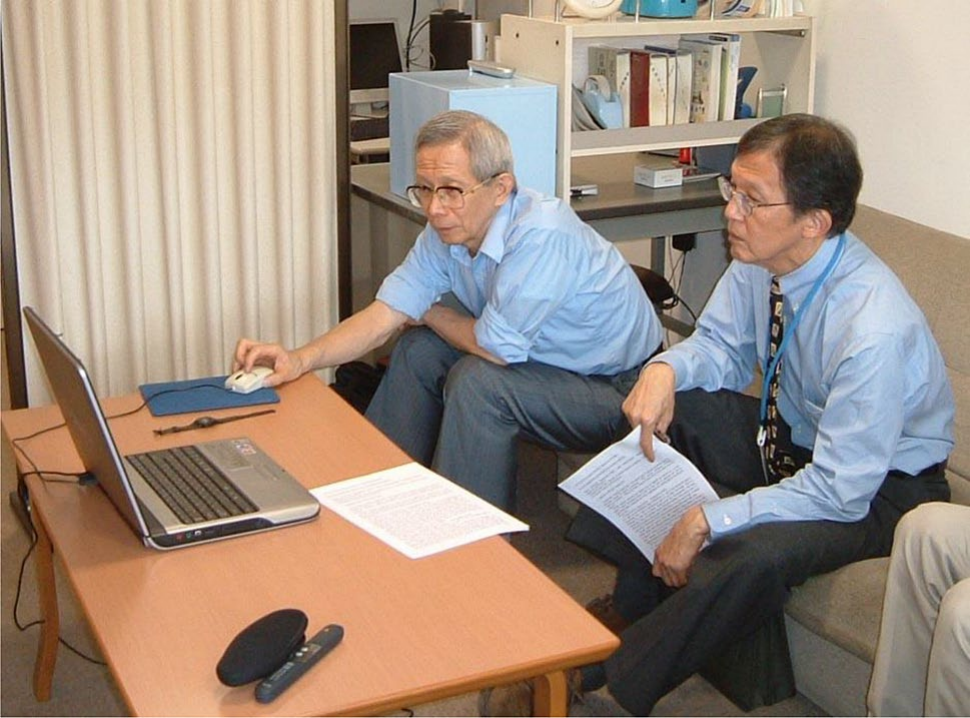
Dr. Aoyagi showing his work to Dr. Byron Aoki of the University of Hawaii (at the author's office at NCCHD, 2002)

Dr. Aoyagi’s explanations and his presentations at conferences had a very unique style using Excel for projection and hand-written explanations. His unique structural style was convincing. You can see this in his invention notes from 1973. Somehow, he reminds me of Maestro Herbert Blomstedt, the 93-year-old conductor (Honorary Conductor Laureate of the NHK Symphony Orchestra) in manner and looks. The Dr. Aoyagi I know outside of work is a person with deep knowledge of music and art. Sometimes he surprised me with his extraordinary knowledge of art history and the arts. He was like a pure young man who threw himself fully into whatever interested him (Fig. 18).

I have a special memory from being with him at a meeting of ISO in September 1987 in Moscow, part of the previous Soviet Union. At the time, the Soviet Union was subject to the ups and downs of perestroika, a bit like what things are like in North Korea now. Dr. Aoyagi arrived at the Moscow airport on a different plane from me and was supposed to give me my visa. But he forgot and had passed through immigration, leaving me in the hands of Soviet immigration for an entire day at the airport. He never realized the seriousness of my situation at the time.

During the same trip, I had a chance to go to the Hermitage Art Museum in St. Petersburg with him. We entered the main hall and he just stood there, back straight, white mask, and not moving. It was rather suspicious drawing the attention of the secret police. He was looking on, blissfully unaware of what was going on. He continued his tour of the museum for several hours happily without noticing the turbulence behind him. Things in Russia are very different now, and it would not be unusual to see an Asian person or even a Russian person in a mask. As far as Aoyagi was concerned, he was just overwhelmed by the sheer number of famous paintings. I was faced with the unlucky task of standing up to the KGB, but in the face of Dr. Aoyagi’s childlike innocence, I could not get myself to try to explain things to him. I will never forget this.

### In closing

Dr. Aoyagi presented his principle of pulse oximeters for the first time at a conference in 1974. The session was chaired by Dr. Tatsuo Togawa (Professor of Medical Engineering at the Tokyo Medical and Dental University), a prominent scientist in the field. Dr. Togawa stated in 2011 that pulse oximeters have developed much more than could be imagined even then from Dr. Aoyagi’s presentation. The possibilities for pulse oximetry using multi-wavelengths are many, including the establishment of a standard method of calibration, improvement in the precision of measurements during low perfusion or body movement, and by including the measurement of other substances or metabolic situations. It may even be possible for it to act like pulse spectrophotometry. This may not be as easy as it sounds to a clinician, but placing our hopes with the scientists following Mr. Aoyagi, I would like to express my gratitude for the great contributions made by Dr. Takuo Aoyagi in this field.

May he rest in peace.
